# Association with the origin recognition complex suggests a novel role for histone acetyltransferase Hat1p/Hat2p

**DOI:** 10.1186/1741-7007-5-38

**Published:** 2007-09-19

**Authors:** Bernhard Suter, Oxana Pogoutse, Xinghua Guo, Nevan Krogan, Peter Lewis, Jack F Greenblatt, Jasper Rine, Andrew Emili

**Affiliations:** 1 Program in Proteomics and Bioinformatics, Banting and Best Department of Medical Genetics, Department of Medical and Molecular Genetics, University of Toronto, Toronto, Ontario, Canada; 2 Department of Molecular & Cell Biology, University of California, Berkeley, CA, USA; 3 Department of Cellular and Molecular Pharmacology, University of California, San Francisco, CA, USA; 4 Department of Biochemistry, University of Toronto, Toronto, Ontario, Canada

## Abstract

**Background:**

Histone modifications have been implicated in the regulation of transcription and, more recently, in DNA replication and repair. In yeast, a major conserved histone acetyltransferase, Hat1p, preferentially acetylates lysine residues 5 and 12 on histone H4.

**Results:**

Here, we report that a nuclear sub-complex consisting of Hat1p and its partner Hat2p interacts physically and functionally with the origin recognition complex (ORC). While mutational inactivation of the histone acetyltransferase (HAT) gene *HAT1 *alone does not compromise origin firing or initiation of DNA replication, a deletion in *HAT1 *(or *HAT2*) exacerbates the growth defects of conditional *orc-ts *mutants. Thus, the ORC-associated Hat1p-dependent histone acetyltransferase activity suggests a novel linkage between histone modification and DNA replication. Additional genetic and biochemical evidence points to the existence of partly overlapping histone H3 acetyltransferase activities in addition to Hat1p/Hat2p for proper DNA replication efficiency. Furthermore, we demonstrated a dynamic association of Hat1p with chromatin during S-phase that suggests a role of this enzyme at the replication fork.

**Conclusion:**

We have found an intriguing new association of the Hat1p-dependent histone acetyltransferase in addition to its previously known role in nuclear chromatin assembly (Hat1p/Hat2p-Hif1p). The participation of a distinct Hat1p/Hat2p sub-complex suggests a linkage of histone H4 modification with ORC-dependent DNA replication.

## Background

In the budding yeast *Saccharomyces cerevisiae*, chromosomal DNA replication during S-phase is initiated from defined origins that are constitutively bound by an evolutionarily conserved multi-protein complex, termed the origin recognition complex (ORC) [1]. ORC is composed of six subunits (Orc1p-6p) that are essential for viability. Its central role is in the initiation of DNA replication, serving as a scaffold for the recruitment of critical initiator proteins such as Cdc6p and Mcm2p-7p that assemble into a pre-replicative complex (pre-RC) in G1 phase (reviewed in [2]). An important determinant of origin firing in S-phase is the binding of key initiation factors such as Cdc45p to autonomous replicating sequences (*ARS*s) just prior to origin firing [3] and melting of the duplex DNA [4]. Whereas ORC remains bound to *ARS *sequences throughout S phase, Cdc45p and other initiation factors such as the minichromosome maintenance (MCM) complex become associated with the advancing replication fork [5,6]. In addition to its well characterized function in mediating the initiation of DNA replication, ORC also contributes to the formation of silent chromatin [7-9]. Moreover, in human cells, additional functions have been reported for ORC subunits in sister chromatid cohesion, chromosome segregation, and cell division [10-13].

Activation of each of the estimated 400 individual origins in the yeast genome occurs at different times in S-phase [14]. The timing and efficiency of origin activation also appears to be dependent on the chromosomal context of a given origin (reviewed in [15]). For instance, in yeast, the repressive heterochromatin structure found near telomeres confers late replication timing to origins [16] Implicating chromatin structure directly in the regulation of DNA replication, the activity of ORC-occupied origins in yeast depends on the nearby orderly positioning of nucleosomes [17,18], This suggests that the local chromatin structure adjacent to *ARSs*, and by inference the proper positioning of nucleosomes and/or the modification states of histones affects origin activation.

Acetylation and deacetylation of the core histones is an important mechanism by which chromatin structure regulates DNA-dependent processes. Most histone acetyltransferases (HATs) identified in yeast to date are components of nuclear multi-subunit protein complexes that remodel chromatin structure [19]. These include Esa1p, Sas2p and Sas3p, which are members of the highly conserved MYST (MOZ, YBF2/Sas3, Sas2, and Tip60) family, and Gcn5p, Elp3p, and Hpa2p, which are members of the GNAT (Gcn5-related N-acetyltransferase) superfamily. Although these HAT complexes, and histone deacetylases (HDACs), control gene expression through modulation of the transcriptional machinery (i.e. promoter usage), increasing evidence suggests that one or more of these complexes may have analogous roles in chromatin remodeling during DNA replication and DNA repair [20-22]. In yeast, deletion of the highly conserved yeast HDAC *RPD3*, which normally deacetylates histones H3 and H4, results in premature origin firing and advanced entry into S-phase [23]. Conversely, artificial tethering of the HAT Gcn5p, which preferentially acetylates nucleosome-bound histone H3, results in earlier origin activation of the late origin *ARS1412 *[23]. Another HDAC, Sir2p, which mediates transcriptional silencing in yeast, has also been implicated in the regulation of pre-RC formation and origin activity [24]. In *Drosophila *follicle cells, the patterns of histone acetylation correlate closely with origin activity and ORC binding [25]. While artificial tethering of the HDAC Rpd3p near to an origin results in reduced firing, recruitment of the *Drosophila *MYST-related HAT Chameau stimulates origin activity. These results imply that regulated acetylation of histone tails likely plays a direct role in regulating origins in eukaryotes. Intriguingly, a human member of the MYST family of HATs, Hbo1p, physically interacts with ORC in tissue culture cells [26,27], suggesting a plausible role for ORC in directing histone acetylation of nucleosomes adjacent to origin DNA.

One of the first discovered yeast HATs, Hat1p, has been linked to histone deposition, nucleosome formation and chromatin assembly during ongoing DNA replication and DNA repair, rather than to a more common function in the regulation of transcription [28-34]. Hat1p was originally identified as a cytoplasmic enzyme that acetylates free, but not chromatin-bound, histone H4 specifically on lysine residues 5 and 12 (K5/12) [30,31]. This pattern of N-terminal acetylation is thought to facilitate the assembly of newly synthesized histones H3 and H4 into chromatin [35] and appears to be evolutionarily conserved, as homologs exhibiting similar target specificity *in vitro *have been detected in other eukaryotes [34,36]. Recently, Hat1p was identified as the catalytic core component of a trimeric nuclear complex with two tightly associated co-factors, namely Hat2p and Hif1p (Hat1p-interacting factor 1; [28,32]). Hat2p, a homolog of the human retinoblastoma binding protein Rba48p, acts as adaptor for high affinity binding of Hat1p to histone H4 [30,31], whereas Hif1p is a histone chaperone [28]. Hif1p is detected exclusively in nuclei, and may accompany newly modified H3–H4 tetramers to facilitate incorporation into nascent chromatin during DNA synthesis. Yeast strains deficient in either *HAT1 *or *HAT2 *exhibit no obvious growth deficiencies [30]. However, gene silencing at telomeres is reduced when *hat1Δ *or *hat2Δ *null alleles are combined with substitution mutations blocking acetylation of the amino terminal tail of histone H3 [29]. This combinatorial effect is phenocopied by combining the same H3 alleles with a lysine 12 to arginine mutation in histone H4 that prevents acetylation, consistent with the known target specificity of Hat1p/Hat2p. Moreover, *hat1Δ *in combination with N-terminal lysine substitution alleles in histone H3 confers hypersensitivity to DNA-damaging agents [33]. Similar phenotypes are recapitulated with deletions in the *HIF1 *gene in combination with H3 tail mutants [28]. Collectively, these data point to the overlapping of acetylation of the N-terminal tail of histone H3 and Hat1p-mediated acetylation of histone H4 in chromosome dynamics.

In extensive synthetic lethal screens that were undertaken to search for novel candidate proteins with potential roles in DNA replication [11], an unexpected genetic interaction between ORC and the *HAT1 *and *HAT2 *genes was uncovered. Here, we describe the identification of a novel Hat1p/Hat2p sub-complex, which is stably bound to ORC, but which lacks Hif1p and is therefore distinct from the Hat1p/Hat2p-Hif1p chromatin assembly complex. Molecular genetic and biochemical studies further indicate functional relationships among Hat1p/Hat2p, ORC, the acetylation of both histones H3/H4, and DNA replication. Collectively, our results suggest a previously overlooked role for a specific conserved HAT in DNA replication in addition to its defined role in chromatin assembly, and point towards partly redundant histone acetylation activities in the control of origin firing.

## Results

### Physical interaction of ORC with Hat1p and Hat2p

**Figure 1:**
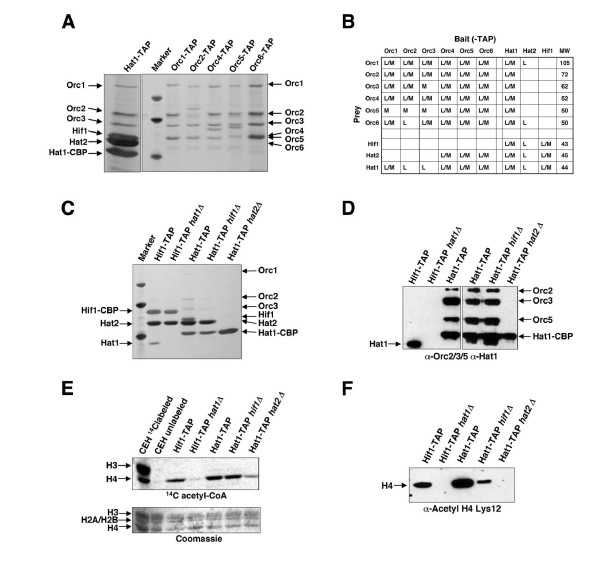
**Physical interaction of ORC with the Hat1p/Hat2p complex**. (A) The B-type HAT complex was purified from a strain containing a TAP-tagged Hat1p subunit, and ORC was purified from strains carrying either a TAP-tagged version of Orc1p, Orc2p, Orc4p, Orc5p, or Orc6p. For comparison, lane 2 shows marker proteins (MWs are 45.0, 66.2, and 97.4 kDa). The positions of the respective subunits of the ORC and Hat1p complex are indicated. Hat1-CBP: Hat1p with calmodulin binding protein (CBP) after TEV digestion of the TAP-tag. (B) Summary of proteins identified in purifications of TAP-tagged baits. Components of ORC and Hat1p complexes that were detected at least once with either MALDI-TOF or liquid chromatography-mass spectrometry (LC-MS) with high confidence (>90%) are indicated (M/L). (C) Architecture of the Hat1p complexes by differential tagging and subunit deletions. Strains were *HIF1-TAP *(BSY675), *HIF1-TAP hat1Δ *(BSY681), *HAT1-TAP *(BSY679), *HAT1-TAP hif1Δ *(BSY720), *HAT1-TAP hat2Δ*(BSY682). Lane 1 shows marker proteins (45.0, 66.2, and 97.4 kDa). (D) Western blot from two series of TAP purifications (Figure 1C for left panel, Additional file 1 for right panel), probed with a-Hat1p, a-Orc2p, a-Orc3p, and a-Orc5p antibodies. (E) *In vitro *histone acetyltransferase activities of Hat1p complexes. Concentrated eluate (10-fold) from indicated TAP-tag purifications from Figure 1C was used for HAT-assays with ^14^C acetyl-CoA and chicken erythrocyte histones. The upper panel shows ^14^C incorporation into histones shown in the lower panel by Coomassie strain. (F) *In vivo *association of acetylated histone H4 with Hat1p sub-complexes from Figure 1C). Eluates from TAP-tag purifications were analyzed for Lys12 acetylated histone H4 by Western blot (a-Acetyl H4 Lys12).

To search for additional proteins that interact with Hat1p, we used the tandem affinity purification (TAP) technique, which has proven to be an effective method for detecting protein complexes in yeast [37,38]. TAP-tagged versions of Hat1p were purified 10^6^-fold to virtual homogeneity from soluble cell extracts derived from large-scale log-phase cultures [39]. As expected, Hat1p co-purified with Hat2p and Hif1p (Figure 1). In addition, and unexpectedly, we detected each of the six subunits of ORC in independent purifications of TAP-tagged Hat1p. The association of ORC was sub-stoichiometric relative to Hat1p, Hat2p and Hif1p, but reproducible and highly specific in that ORC was detected in only a few other successful purifications of over 2500 different yeast proteins [37].

**Figure 2:**
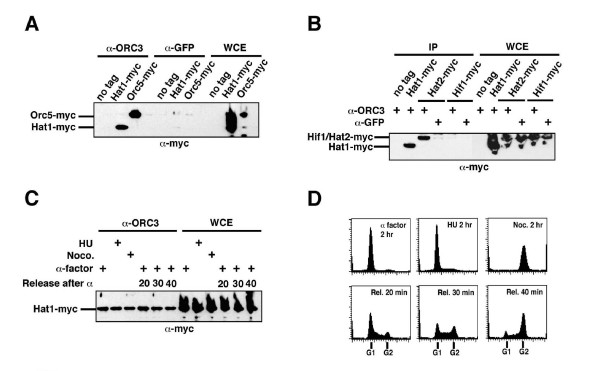
**The constitutive association of Hat1p/Hat2p with ORC**. (A) Co-immunoprecipitation (IP) of Hat1p with ORC from log-phase cells. Strains were: untagged control (JRY2334), Hat1–13myc (BSY676), and Orc5–13myc (BSY677). IPs were performed with monoclonal a-Orc3p and a-GFP antibodies. One twentieth of the extracts were loaded as whole cell extract (WCE). Immunoprecipitated Hat1–13myc was detected with mouse α-myc as a primary antibody (9E10). Note that equal amounts and concentrations of extracts were used for all IPs. (B) ORC binds Hat1p/Hat2p but not Hif1p. Strains were: untagged control, Hat1–13myc, Hat2–13myc (BSY691), and Hif1–13myc (BSY692). One twentieth of the extracts were loaded as WCE control. IPs were performed with monoclonal α-Orc3p and α-GFP control antibodies. (C) IP of Hat1p-13myc with α-Orc3p antibody in synchronized cells. Log-phase cells were either arrested in 3 μg/ml α-factor, 200 mM hydroxyurea, or 15 μg/ml nocodazole. Cells were also released from α-factor arrest and samples were taken at 20, 30, and 40 min after release (lanes 4–6). Lanes 7–12 show WCE controls. (D) FACS control of samples in panel C. Time after release is indicated (Rel).

As ORC has not been found previously to interact with any member of the Hat1p complex, we performed stringent reciprocal affinity tagging and purification experiments using C-terminally TAP-tagged versions of ORC subunits (Orc1p-6p). In addition to all known subunits of the ORC complex, Hat1p and Hat2p were often detected, albeit again sub-stoichiometrically and not easily visible by silver stain (Figure 1A,B). We independently tested the ORC-Hat1p interaction by immunoprecipitation of an endogenously myc-tagged version of Hat1p using a monoclonal antibody specific for Orc3p (Figure 2). Co-immunoprecipitation with this antibody led to the detection of myc-tagged Hat1p as seen with myc-tagged Orc5p (Figure 2A), whereas these proteins were not detected in immunoprecipitations with an anti-GFP control antibody. When we tested for the association of Hat2p and Hif1p with ORC using an ORC3-specific antibody, we found that ORC interacts with Hat2p but not Hif1p (Figure 2B). Importantly, co-immunoprecipitation of Hat1p with ORC was also observed when precipitated proteins were treated with the DNA-degrading enzyme benzonase (Additional file 1).

To further define Hat1p complex architecture, mainly with respect to its association with ORC, we subsequently performed purifications of TAP-tagged Hat1p and Hif1p with other subunits deleted (Figure 1C, Additional file 1). Notably, Hat1p was observed in sub-stoichiometric amounts when Hif1-TAP was purified whereas the Hat2p/Hif1p association was unaffected by *HAT1 *deletion. Given that the association of Hat1p/Hat2p with ORC was only weakly visible by SDS-PAGE/silver staining gel, concentrated purification products were analyzed by Western blot using antibodies against the Orc2p, Orc3p, and Orc5p subunits (Figure 1D). Upon repeated purification of Hif1-TAP, the Hat2p and Hat1p subunits were identified, but ORC was conspicuously absent (Figure 1C,D). Importantly, we also found that ORC remains associated with Hat1p/Hat2p even in a strain where *HIF1 *is deleted, whereas loss of Hat2p leads to disintegration of the entire complex (Figure 1D). Collectively, our analysis confirms a specific, and previously overlooked, physical interaction between ORC and a Hat1p/Hat2p sub-complex that is distinct from the established nuclear Hat1p/Hat2p-Hif1p.

To assess the enzymatic functions of the purified complexes, we assayed the transfer of ^14^C-labeled acetyl-CoA by the Hat1p sub-complexes onto purified core histones from chicken erythrocytes (Figure 1E, Additional file 1). In agreement with previous findings [30,31], we found that histone acetyltransferase activity by Hat1p was enhanced approximately 10-fold in the presence of Hat2p. Substrate specificity and activity on histones, however, seemed not to be altered by the presence of Hif1p and ORC. When we tested for the *in vivo *association of Lys12-acetylated histone H4 by Western blot, we found that it is largely dependent on intact Hat1p complex (Figure 1F). The absence of Hat1p or Hat2p leads to a complete loss of acetylated histone H4 from the residual complex components, implying both of these factors in the interaction with acetylated H4.

Considering the role of ORC in the formation of the pre-RC during G1 phase, we next examined if the association of Hat1p with ORC was cell-cycle dependent. We performed co-immunoprecipitations using whole cell extracts prepared from yeast cultures arrested either in G1 phase with α-factor, in S-phase with hydroxyurea, or in G2/M with nocodazole, as well as from cells synchronously released into S-phase after an α-factor imposed G1 block (Figure 2C). Surprisingly, a comparable level of myc-Hat1p co-precipitated with ORC throughout these cellular treatments, suggesting that Hat1p, and by inference the Hat1p/Hat2p sub-complex, remained stably associated with ORC throughout the cell cycle. Consistent with this finding, large-scale purifications and mass spectrometric analysis of TAP-tagged Orc2p from α-factor arrested cells confirmed the association of Hat1p/Hat2p with ORC in the G1 phase (data not shown). By contrast, we failed to detect components of the pre-RC (such as Cdc6p) under these same purification conditions (BS, unpublished observations). The pre-RC could be readily disrupted, whereas the Hat1p/Hat2p-ORC interaction may be physically more robust under the chosen experimental conditions.

### Genetic interaction between *orc-ts *and *hatΔ/hat2Δ *mutants

Previously, genome-scale screens using the synthetic genetic array (SGA) methodology were performed to identify novel functional partners of ORC [11]. An array of about 4600 haploid deletions was combined with conditional temperature-sensitive (*ts*) *orc2-1 *and *orc5-1 *alleles which are hypomorphic for initiation of DNA replication, and scored for slow growth or lethality of the double-mutant meiotic progeny. We repeatedly detected genetic interactions (enhanced slow growth or synthetic sickness), leading to a reduced maximum permissive temperature, between either *orc2-1 *or *orc5-1 *and *hat1 *or *hat2 *deletions. These functional associations were highly specific as few other interactions with *hat1 *or *hat2 *were identified in SGA screens of over 200 unrelated query genes [40].

**Figure 3:**
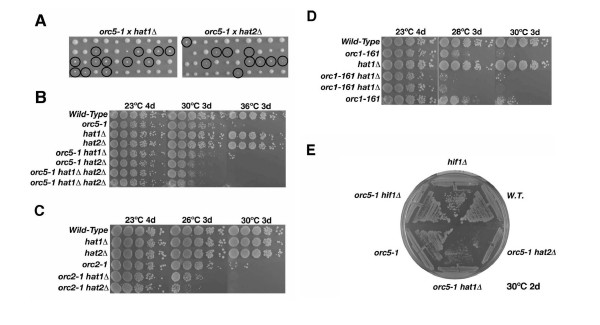
**A genetic interaction between *orc-ts *alleles and mutations in the B-type histone acetyltransferase**. (A) Analysis of tetrads from a cross of *orc5-1 *(JRY4250) with *hat1Δ *(BSY539) and *hat2Δ *(BSY540). Spores were incubated for 3 days at 31°C, which is the semi-permissive temperature for *orc5-1*. Double mutants are marked with circles. (B) Plating assay of *orc5-1 hat1Δ *(BSY538), *orc5-1 hat2Δ *(BSY602), and *orc5-1 hat1Δhat2Δ *(BSY603, BSY604) with control strains at indicated times and temperatures. Dilutions were 1:10, starting from late log-phase cultures on YPD. (C) Plating assays for *orc2-1 *mutant combinations with *hat1Δ *(BSY569) and *hat2Δ *(BSY572). (D) Plating assays as in B for *orc1-161 *mutant combinations with *hat1Δ *(BSY589-BSY591). (E) Growth comparison of *orc5-1 hif1Δ *(BSY595) with *orc5-1 hat1Δ *and *orc5-1 hat2Δ *at 30°C. For a detailed list of strains, see Additional file 7.

The synthetic composite phenotype of *hat1 *and *hat2 *in combination with *orc5-1 *and *orc2-1 *was independently confirmed by tetrad analysis in the *W303 *genetic background (Figure 3). Deletions of either *HAT1 *or *HAT2 *markedly reduced the growth rate of *orc5-1 *mutant cells at the semi-permissive temperature of 31°C. The double mutants formed small, slower growing colonies (Figure 3A). To exclude the possibility that the reduced colony size of the double mutants was caused by a delay in germination, the synthetic growth defect of *orc5-1 hat1Δ *and *orc5-1 hat2Δ *mutants was further confirmed by a serial dilution assay of mitotically dividing cells. While the double mutants were fully viable at 23°C, a loss of viability was observed at 31°C (Figure 3B), which is the maximal permissive temperature for the *orc5-1 *allele. Combination of *orc2-1 *or *orc1-161 *with either *hat1Δ *or *hat2Δ *also reduced growth at the maximum permissive temperatures of 26°C and 28°C (Figure 3C,D). The *hat2Δ *mutation caused a slightly greater slow-growth phenotype than *hat1Δ *when combined with either the *orc2-1 *and *orc5-1 *alleles. The reason for this subtle distinction is presently unclear, but could be an indication of redundant HAT activities. However, no further reduction of viability was observed in *orc5-1 hat1Δhat2Δ *triple mutants, consistent with a virtually complete functional (i.e. epistatic) overlap between Hat1p (catalytic core) and Hat2p (histone targeting co-factor). By contrast, deletion of *HIF1 *did not lead to a reduction of the permissive temperature for *orc5-1 *mutations (Figure 3E), pointing again to the absence of a functional interaction between Hif1p and ORC. As synthetic genetic interactions with conditionally lethal mutations are often indicative of a shared or overlapping biological function, these data suggest that Hat1p/Hat2p likely shares a function with ORC, presumably in DNA replication, separate from its function with Hif1p in chromatin assembly [28]. However, we observed only a very subtle reduction of colony size when *hat1 *was combined with the replication defective mutant *mcm2-1 *(Additional file 2), while virtually no interaction was detectable between *hat1 *or *hat2 *null alleles in combination with *ts*-alleles in other essential initiation factors examined (e.g. *cdc7-1 *or *cdc6-1*; Additional file 2 and data not shown). Hence, our data suggest that the genetic interaction observed between *orc-ts *mutations and *hat1Δ/hat2Δ *is specific and closely mirrors the physical interaction.

### *HAT1 *mutations affect cell viability and cell-cycle progression in *orc5-1 *mutants

**Figure 4:**
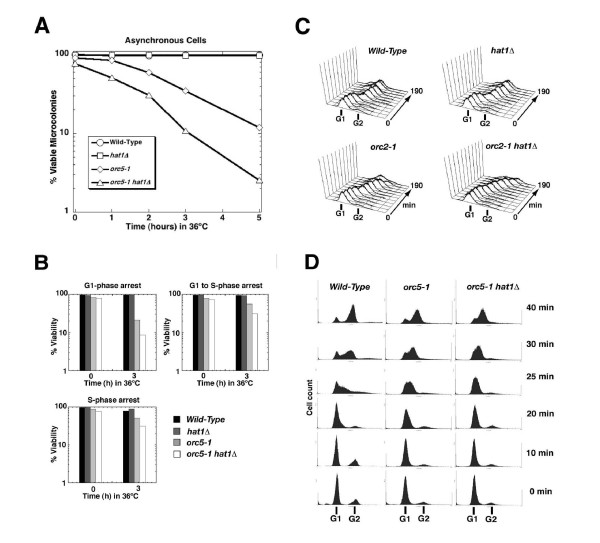
**The *hat1Δ *mutation reduces viability and enhances the cell-cycle defect of *orc-ts *cells**. (A) Percentage of cells that form viable microcolonies when asynchronous cultures of *orc5-1 hat1 *(BSY538), *orc5-1 *(BSY535), *hat1 *(BSY528) and wild-type strain (JRY2334) were shifted to the nonpermissive temperature for 0–5 h. Viability was measured as the fraction of microcolonies that formed after the incubation at 36°C within 1–2 days at permissive temperature. (B) Percentage of *orc5-1 hat1, orc5-1*, *hat1*, and wild-type cells that form viable microcolonies when synchronized cultures were shifted to the nonpermissive temperature. Cells were arrested in G1 (α-factor) or in S-phase (hydroxyurea) at 23°C and then maintained at restrictive temperature (36°C) for 0 to 3 h in G1 phase, in S-phase or from G1 to S-phase arrest. Averages of two independent experiments are shown. (C) FACS analysis of wild-type, *hat1Δ *(BSY539) *orc2-1 *(BSY568) and *orc2-1 hat1Δ *(BSY569) cells at semi-permissive temperature (26°C). Cells were arrested in α-factor (5 mg/ml) and release was performed at 26°C for 0, 10, 20, 30, 40, 50, 60, 90, 120, 150, and 190 min. (D) Cell-cycle progression of *orc5-1 hat1, orc5-1 *and wild-type strain at restrictive temperature for *orc5-1*. G1-arrested cells were held at 36°C (restrictive temperature) for 1 h and then released into fresh (36°C) medium. Samples for FACS were taken at times indicated. For a detailed list of strains, see Additional file 7.

To quantitatively monitor the loss of viability, asynchronous cultures of *hat1Δ*, *orc5-1 *single, and *orc5-1 hat1Δ *double mutants were shifted to the restrictive temperature for *orc5-1 *(36°C) for up to 5 h (Figure 4A). Samples were taken at distinct timepoints and the fraction of cells capable of forming viable microcolonies upon rescue to room temperature was measured. In this manner, we confirmed that *hat1Δ *indeed enhanced the loss-of-viability of *orc5-1 *alleles held at 36°C. By contrast, *hat1Δ *single mutant cells retained full viability as compared to the wild-type control, indicating that the *hat1Δ *loss-of-function mutation specifically amplifies the viability defects associated with impaired ORC. When synchronized cells were shifted to the restrictive conditions, *orc5-1 *cells were distinctly more sensitive to inactivation in G1 relative to S-phase (Figure 4B), reflecting the major role of ORC in pre-RC formation in G1 and being consistent with previous findings for *orc2-1 *[41]. Deletion of *HAT1 *enhanced this effect approximately twofold.

Growth arrest of *orc2-1 *and *orc5-1 *strains occurs predominantly at the G2/M transition [7,9], and is dependent on activation of the DNA damage and spindle surveillance checkpoints in response to impaired or incomplete DNA replication [42]. As a measure of any further compromise in DNA replication efficiency, we examined whether the G2/M transition was additionally delayed in *orc2-1 hat1Δ *strains using flow cytometry (Figure 4C). Log-phase cultures of the single and double mutants and the wild-type controls were arrested with α-factor, and then synchronously released into the cell cycle at about 26°C, which is semi-permissive for *orc2-1*. Partial accumulation of *orc2-1 *cells in G2/M was readily observed following completion of the bulk of DNA synthesis (around 130 min), as expected from previous studies [43]. However, the fraction of *orc2-1 hat1Δ *double mutants that remained permanently arrested in G2/M at 26°C was substantially larger than that seen with the *orc2-1 *single mutant alone (Figure 4C). A similar enhancement of the *orc-ts *phenotype was observed in combination with the *orc5-1 *allele (see Additional file 3). By contrast, the overall cell-cycle progression profiles of the *hat1Δ *and *hat2Δ *single mutant strains were unperturbed, and comparable to a wild-type control (Figure 4C, Additional file 3). Consistent with inactivation of origins by *orc-ts *mutations resulting in a DNA damage response, we detected faster cell-cycle progression of *orc5-1 *single and *orc5-1 hat1Δ *double mutants after inactivating the *RAD9 *and *RAD24*-dependent DNA damage checkpoint pathways (see Additional file 3). In addition to the impaired G2/M transition, the consequences of inactivation of origins in *orc5-1 *strains were manifested by impaired S-phase progression at the fully restrictive temperature of 36°C (Figure 4D). Flow cytometric analysis showed that completion of DNA synthesis took longer in *orc5-1 *mutants as compared to wild-type cells. Importantly, this delay in S-phase progression became even more pronounced in the *orc5-1 hat1Δ *double mutants (see profiles at 25 and 30 min), consistent with the loss of *HAT1Δ*further compromising the DNA replication and cell-cycle defects stemming from the *orc5-1 *conditional allele. Hence, the shared Hat1-ORC function appeared to reflect some core aspect of ORC-driven DNA replication.

### Hat1p localizes to firing origins and persists with replicating DNA during S-phase

**Figure 5:**
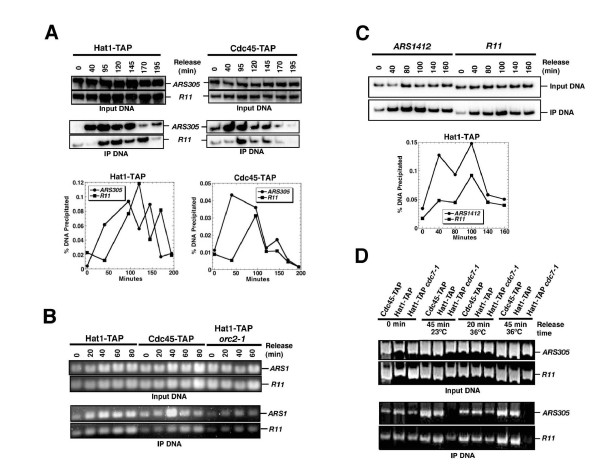
**Hat1-TAP is recruited to early and late origins at the time of firing**. Samples were taken at indicated times and processed for chromatin immunoprecipitation and input controls. DNA was either labeled with ^32^P (panels A and C) or visualized by ethidium bromide (panels B and D). (A) Binding of Hat1-TAP and Cdc45-TAP to *ARS305 *and to *R11 *control sequence at the time of origin firing and later in S-phase. Strains were *HAT1-TAP *(BSY679) and *CDC45-TAP *(BSY680). One representative experiment with input and immunoprecipitate and the percentage of precipitated DNA is shown. (B) Recruitment of Hat1p to *ARS1 *is coincident with Cdc45p and dependent on functional ORC. Strains used for chromatin immunoprecipitation are *HAT1-TAP*, *CDC45-TAP*, and *HAT1-TAP orc2-1 *(BSY699). Strains were held in a-factor at 36°C to inactivate *orc2-1 *and then released at 23°C. (C) Recruitment of Hat1p (Hat1-TAP) to *ARS1412 *late replication origin and comparison with *R11 *sequence. (D) Recruitment of Hat1p to *ARS1 *is affected by the *cdc7-1 *allele. Strains used for chromatin immunoprecipitation are *HAT1-TAP*, *CDC45-TAP*, and *HAT1-TAP cdc7-1 *(BSY734).

As ORC recruits replication initiation factors to origins [44], we examined if Hat1p associates with origin DNA, either throughout the cell cycle (such as ORC, and as might be expected for a stable Hat1p/Hat2p-ORC complex) or specifically during S-phase (such as Cdc45p). To this end, we performed chromatin immunoprecipitation (ChIP) assays to measure direct binding of TAP-tagged Hat1p to selected *ARS *sequences and control DNA at various timepoints after release into S-phase from α-factor-mediated G1 arrest (Figure 5). Parallel analysis was performed with strains containing TAP-tagged versions of Cdc45p as positive controls. ORC is bound exclusively to origin DNA, whereas Cdc45p, after being recruited to origins, associates with the advancing replication fork upon origin firing [5,6]. We measured binding to two early origins, *ARS305 *and *ARS1*, and to late origin *ARS1412*, as well as to the control sequence *R11 *that is not known to be immediately adjacent to any active origin. To improve overall resolution, S-phase progression was slowed by release of the synchronized cell population into growth medium at 16°C or 20°C.

While no or only minor binding of Hat1p-TAP to *ARS *sequences was observed immediately at α-factor arrest (0 min timepoint), Hat1p-TAP bound specifically to *ARS305 *after release into S-phase. (Figure 5A). Peak *ARS *binding of Hat1p coincided with that of Cdc45p, and by inference with origin activation, and was followed by diminished binding at later timepoints, concurrent with the completion of initiation. An increase of immunoprecipitate was detected at the *R11 *control sequence with a lag relative to *ARS305*. An enrichment of Hat1p during S-phase, similar to that of Cdc45p, was also evident with *ARS1 *(Figure 5B). The association of Hat1p-TAP with the late firing *ARS1412 *was virtually indistinguishable from the binding to *R11*, consistent with late activation and replication timing of this origin (Figure 5C). Thus, our data indicate that Hat1p becomes associated with origins of replication around the time of origin firing and later with non-origin sequences, such as Cdc45p, suggesting that Hat1p also associates with advancing replication forks. No recruitment of Hat1p to *ARS1 *and *R11 *was observed when DNA replication was compromised by shifting the *orc2-1 *allele to the restrictive temperature (36°C). However, association with *ARS1 *is similarly abolished or lowered by the *cdc7-1 *mutation at restrictive (36°C) and at permissive (23°C) temperature (Figure 5D). As *cdc7-1 *did not show a genetic interaction with *hat1Δ*, the recruitment of Hat1p depends on DNA replication in general but not specifically on intact ORC. Importantly, the dynamic recruitment of Hat1p to origins during S-phase contrasts also with the constitutive association of Hat1p/Hat2p with ORC as seen by co-immunoprecipitation (Figure 2C). Hence, the discrepancies in our data can at least partly be explained by the existence of two separate complexes, Hat1p/Hat2p-ORC and the previously reported Hat1/Hat2-Hif1p. The S-phase specific recruitment of Hat1p could reflect the documented role in chromatin assembly during fork progression mediated by the Hif1p-containing complex [28].

**Figure 6:**
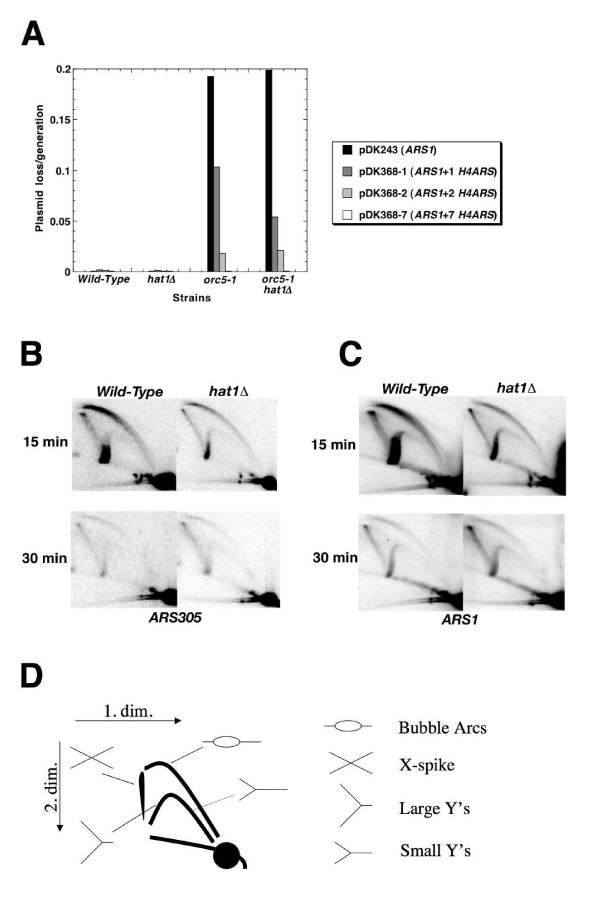
**Analysis of DNA replication phenotypes**. (A) Plasmid loss rates were measured in *orc5-1 hat1*, *orc5-1, hat1*, and wild-type control strains by growing the cells for approximately 10 generations. Strains were JRY2334 (*W303 *wild-type control), BSY528 (*hat1*), BSY535 (*orc5-1*), BSY538 (*orc5-1 hat1*). These strains were transformed with plasmids pDK243, pDK368-1, pDK368-2, pDK368-7, containing 1, 2, 3 or 8 *ARS *sequences, respectively. Y-axis shows plasmid loss rates (loss frequency/generation). (B,C) Structural analysis of replicative intermediates by two-dimensional gel electrophoresis. Cultures of *hat1 *(BSY528) and wild-type (JRY2334) strains were released from G1 arrest for 15 and 30 min (30°C). The same blot was first hybridized with an *ARS305 *specific probe (B), then stripped and rehybridized with an *ARS1 *specific probe (C). The 15 min wild-type sample is overloaded as judged by the amount of monomers. (D) Scheme for replicative intermediates observed in two-dimensional gel electrophoresis.

A replication defect in the absence of Hat1p may be expected in case Hat1p/Hat2p-ORC contributes to the function of an origin. To understand whether there was a contribution of Hat1p/Hat2p to the function of a specific origin (*ARS1*), we made use of a plasmid-based minichromosome maintenance assay, which is often used to characterize mutations that affect the efficiency of initiation of DNA replication [45]. Maintenance of a single *ARS1*-containing plasmid was largely perturbed at the restrictive temperature in *orc5-1 *mutants (Figure 6A), but rescued by the presence of multiple *ARSs*. Multiple *ARSs *also suppressed the plasmid loss defect seen with the *orc5-1 hat1 *double mutants; however, this strain was not any more defective in plasmid maintenance in comparison with the *orc5-1 *single mutant. Moreover, the rate of plasmid loss in *hat1Δ *single mutants was not elevated relative to wild-type cells. Thus, Hat1p seems not to have an effect on initiation frequency and DNA replication of the widely studied origin *ARS1*.

To allow for detection of incomplete or defective replication intermediates, or of altered timing of chromosomal origin function, we investigated the effect of deleting *HAT1 *on the activity of specific chromosomal origins (*ARS305 and ARS1*) using neutral two-dimensional gel electrophoresis. For DNA extraction, we performed the cetyltrimethylammonium bromide (CTAB) method, developed by Lopes and colleagues [46], The level of active origin firing was assessed by comparing active replication intermediates (bubble arcs) to the amount of monomeric DNA, whereas passive replication was reflected by the relative amounts of detectable small Y-form molecules (Figure 6D, calculations not shown). However, no distinct alterations in any replicative intermediates were detectable at either of several early (*ARS1, ARS305*) or late (*ARS603, ARS1412*) origins tested in a *hat1Δ *single mutant background (Figure 6B,C, and data not shown). Similarly, no obvious effect on replication initiation (*ARS1, ARS305*) was observed when replicative intermediates are compared between *orc5-1 *and *orc5-1 hat1 *(Additional file 4). The absence of a direct role of Hat1p in the efficiency and timing of these origins could be explained by the possibility that Hat1p/Hat2p does not interact with ORC at these origins and that the S-phase recruitment of Hat1p is solely mediated by Hat1p/Hat2p-Hif1p. However, crosstalk between the two complexes may also occur and other plausible explanations for these observations cannot be dismissed (see Discussion).

Is it possible that Hat1p/Hat2p-ORC highlights a distinct function of ORC that is different from its role in DNA replication? A pertinent function in chromosome metabolism may be related to the role of ORC in transcriptional silencing that is genetically separable from its role in DNA replication [47]. The N-terminal portion of Orc1p is nonessential and therefore dispensable for DNA replication, but is required for the ORC silencing function [48]. This defect can be functionally substituted by the N-terminus of Sir3p, which shares sequence similarity with the Orc1p N-terminus. However, we observed that myc-tagged Hat1p still co-precipitated with ORC in a strain bearing a N-terminal truncation of Orc1p or the N-terminus of Sir3 (Additional file 5). From this, the Hat1p/Hat2p-ORC association seems not to be dependent on the silencing function by the Orc1p N-terminus.

### Histone H4 acetylation is associated with yeast ORC

**Figure 7:**
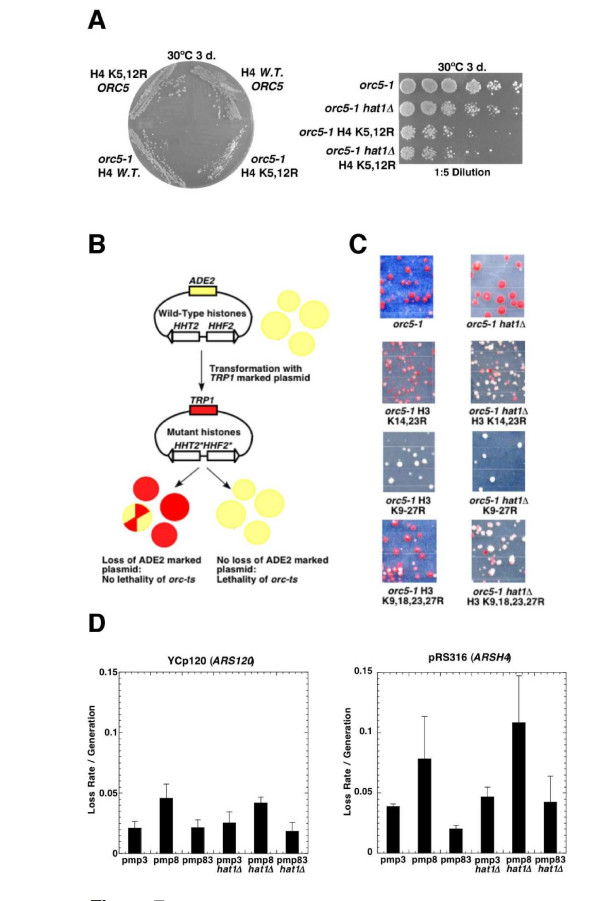
**Growth defect of *orc5-1 *ts in combination with histone tail mutants**. In a plasmid shuffle assay, *orc5-1 *strain (AP121) and *orc5-1 hat1 *strain (AP123) were transformed with *TRP-*marked plasmids that contain histones H3 and H4 with lysine to arginine substitutions at different sites in their N-terminal tails. (A) Arginine substitution in H4 lysine 5, 12 shows reduced viability in combination with *orc5-1 *on YPD at the temperature indicated (31°C). Cells were grown for 3 days. (B) The replication defect in *orc5-1 *leads to efficient loss of the *ADE2 *marked plasmid with the covering H3/H4 wild-type genes when selection is performed on -TRP medium. When the combination of *orc5-1 *with histone mutants is nonviable, cells are less likely to lose the covering plasmid. Colonies were grown at 23°C. (C) Direct assays of transformation and loss of wild-type histone plasmid in different H3 mutant backgrounds. H3 histone mutants contain arginines substituted for lysines. (D) Plasmid loss assays with strains (AP182, AP183) containing wild-type histone H3 (pmp3), K9, 14, 18, 23, 27R substituted histone H3 (pmp8), and K14, 23R substituted histone H3 (pmp83). Loss rates were measured for *ARSH4 *(pRS316) and *ARS120 *(YCp120). Averages and standard deviations of three independent experiments are shown.

As Hat1p/Hat2p has been implicated in a specific pattern of histone acetylation [30,31], we next determined if the relevant role of Hat1p with regard to ORC function lies in its targeted acetylation of lysine residues 5 and 12 on histone H4. Specifically, we tested if non-acetylatable versions of histone H4 that mimic the deacetylated state would exhibit an enhanced phenotypic effect in conjunction with impaired ORC function (Figure 7). To this end, we created a strain expressing a conditional *orc5-1 *allele into cells in which the only source of histone H4 is a modified form of the histone H4 gene encoding residues 5 and 12 mutated to non-acetylable arginines. This variant was introduced along with other selected control histone mutants into either *orc5-1 *or *orc5-1 hat1Δ *strain backgrounds. When combined with *orc5-1*, non-acetylable H4 lysines 5 and 12 resulted in a noticeable reduction in growth (Figure 7A, Additional file 6), indicating that acetylation of these residues is indeed functionally relevant with respect to ORC activity. The lack of a complete epistatic effect (i.e. lysines 5 and 12 mutations conferred a stronger effect than *hat1Δ*) could reflect that histone H4 is a target of additional HATs such as Esa1p and Gcn5p [49,50].

The N-terminal tails of histones H3 and H4 have been shown to be functionally interchangeable [51,52]. To determine if a partly redundant HAT complements Hat1p with regard to ORC function, we first examined if the viability of *orc-ts hat1Δ *double mutant cells was dependent on native (i.e. fully functional) histone H3. Using a plasmid shuffle assay, we generated various combinations of N-terminal lysine substitution mutations in histones H3 in the *orc5-1 *and *orc5-1 hat1Δ *mutant backgrounds bearing an *ADE2*-marked wild-type histone construct (Figure 7B). The ability of the strains to tolerate the mutant histone derivatives was then scored visually based on the rate of loss of the *ADE2 *marked plasmid (sectoring) when no selection was applied. While a control *TRP1*-marked wild-type histone H3 plasmid readily supported growth in *orc5-1 *and *orc5-1 hat1Δ *strains, a histone H3 multi-point mutant lacking acetylatable N-terminal lysines (K9, 14, 18, 23, 27R) conferred a severe loss of viability as indicated by the exclusively white colony color (Figure 7C). Likewise, *orc5-1 *H3 (K9, 14, 18, 23, 27R) mutants were completely inviable when shifted at 30°C (see Additional file 6). Interestingly, mutations of lysine residues 14 and 23 in histone H3 enhanced the growth defects of *orc5-1*, and were incompatible with *orc5-1 hat1Δ *double mutants (Figure 7C, Additional file 6). Taken together, these results indicate that the functional overlap between H3 acetylation and Hat1p-mediated acetylation of H4 likely extends to the ORC-dependent process.

We next examined if *hat1Δ *combined with a non-acetylatable histone H3 mutant tail leads to a plasmid loss phenotype. Plasmid loss rates were measured in wild-type and *hat1Δ *strains using both a plasmid bearing the late replicating telomeric origin *ARS120 *(YCp120) or the early replicating origin *ARSH4 *(pRS316) in combination with either wild-type histone H3 or one of two multi-point mutants H3 (K9, 14, 18, 23, 27R) and H3 (K14, 23R). Elevated loss rates were observed for both plasmids in strains expressing a fully non-acetylatable H3 variant (Figure 7D), a situation that compromised growth of the cells (Additional file 6). However, no additional or synergistic defect was detected when the histone mutants were further combined with the *hat1Δ *mutation. Although this experiment did not establish a replication function for Hat1p at these two origins, it nevertheless showed that plasmid loss occurs below a threshold level of core histone acetylation. While this effect may be an indirect consequence of the histone H3 mutations on transcription or other pathways, it is also consistent with the view that multiple HAT activities converge to maintain genomic integrity.

**Figure 8:**
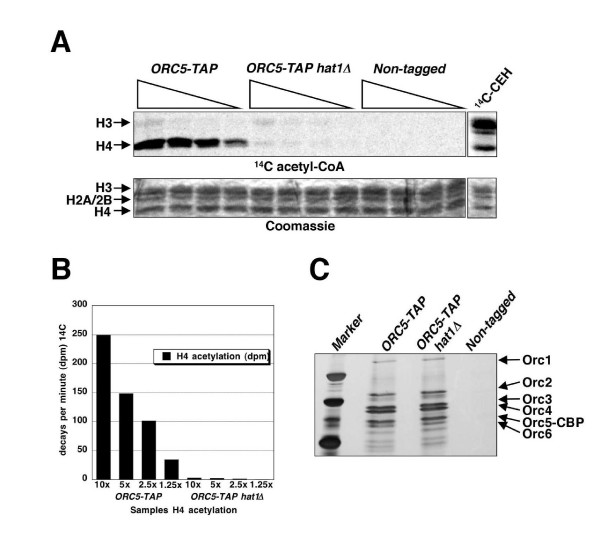
**ORC-associated histone acetylation**. (A) Incorporation of ^14^C-AcCoA into core histones from chicken erythrocytes by ORC-associated histone acetyltransferase. The upper panel shows PhosphorImage of ^14^C-labeled histones and the lower panel shows Coomassie stained gel. Protein introduced in the assay was 10, 5, 2, and 1.25 ml of the TAP-purified and concentrated product (approximately 100 ml). (B) Quantification of the results in panel A. Disintegrations/decays per min are shown on the Y-axis. (C) Equal volumes of TAP-purified proteins used for the HAT assay from *ORC5-TAP *(BSY700), *ORC5-TAP hat1Δ *(BSY701), and untagged control strain (JRY2334) are loaded on a silver stain gel.

The functional connection between ORC and histone H4 acetylation was further corroborated by examining the acetyltransferase activity of affinity purified ORC towards histone H4 *in vitro *(Figure 8). Comparison of the relative activity of *ORC5-TAP *purified from a wild-type strain with that obtained from a *hat1Δ *mutant demonstrated that nearly all histone H4 HAT activity (> 90%) was dependent on Hat1p (Figure 8A,B). Nevertheless, the residual H4 and some possible H3 acetylation in the absence of Hat1p suggests that at least one alternate HAT associates with ORC, albeit more weakly. Thus, whereas the H4-specific HAT activity by Hat1p associated with ORC established a direct connection between ORC function and histone acetylation, additional evidence also suggested the existence of one or more partly redundant, yet unidentified, HAT enzymes that seemingly function in conjunction with ORC.

## Discussion

Hat1p is somewhat unusual in that, unlike most of the other yeast HATs, it has not been found in association with larger multimeric complexes regulating transcription. Rather, it was first detected as a cytoplasmic B-type enzyme specific for free, but not nucleosome-bound, histone H4 [30] and, to a lesser extent, histone H2A [28,34], suggesting a major role in the basal acetylation of newly synthesized histones. However, Hat1p is now known to be the core component of a nuclear localized protein complex [28], together with the cofactors Hat2p and Hif1p. Hat1p-mediated acetylation of K5,12 on histone H4, an epigenetic mark which precedes DNA replication and which is rapidly removed after incorporation into DNA, is highly conserved throughout eukaryotic evolution [35]. Recently, the Hat1p/Hat2p-Hif1p complex was shown to be recruited to DNA double strand breaks, concomitantly with Rad52p [53], suggesting that Hat1p is responsible for histone modifications during DNA repair, thus explaining the phenotypes observed earlier [33]. In this study, we establish that a dynamic recruitment of Hat1p also occurs on chromatin in S-phase. The Hat1p association with origin DNA is analogous to that observed previously with a well established initiation cofactor, Cdc45p, which also advances with replication forks after recruitment to and release from an activated origin [5,6]. This implies that a nuclear Hat1p sub-complex, possibly containing Hif1p in addition to Hat2p, follows the advancing replication fork, consistent with a role in chromatin assembly during ongoing DNA synthesis [28].

**Figure 9:**
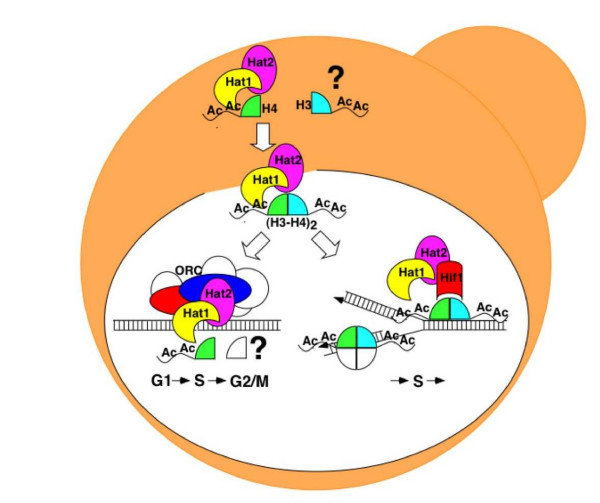
**The current view on the different associations of Hat1p within the cell**. In addition to the cytoplasmic Hat1p/Hat2p complex, the Hat1p/Hat2p-Hif1p chromatin assembly complex is recruited to chromatin during S-phase. A minor fraction of Hat1p/Hat2p is constitutively associated with ORC and presumably chromatin-bound. Additional H3-specific acetylation and additional substrates for Hat1p are possible.

In addition to histone maturation in the cytoplasm and nucleosome assembly in the nucleus, our data now suggests an additional role for Hat1p through a direct interaction with ORC (Figure 9) that is distinct from its role as a subunit in the Hif1p-dependent chromatin assembly complex. The previously described roles of Hat1p and Hat2p in telomeric gene silencing and double-strand break-repair involved also the Hif1p chromatin assembly factor [28]. Like the association of ORC subunits with chromatin, the interaction of ORC with Hat1p is constitutive during the cell cycle. Based on the cumulative evidence gathered so far, we presume that Hat1p and Hat2p may enhance ORC function by contributing either to the overall efficiency of origin usage or through alteration of the chromatin structure of a specific subset of origins, most likely through acetylation of lysine residues 5 and 12 on histone H4. Although we have not identified the exact mechanism involved, the description of a Hat1p/Hat2p-ORC complex is consistent with an emerging expansive view of histone modifications as critical determinants of DNA metabolism [15,23,24].

Considering that ORC function has been studied extensively both in yeast and metazoa, it is perhaps somewhat surprising that the ORC-HAT interaction was not detected before. Most likely, this may be due to limitations of the more traditional biochemical procedures that were used for the purification of ORC and Hat1p. The affinity purification procedure used in this study differs from an analogous method reported previously [38] in that the ion buffer concentrations were lower, preserving the stability of many larger assemblies [39]. In purifications of ORC-TAP, the association of Hat1p was consistently sub-stoichiometric, suggesting that most ORC is not bound to Hat1p/Hat2p. Conversely, ORC is also clearly less abundant than Hat1p/Hat2p, implying that more of Hat1p is associated with Hif1p than with ORC. Is it possible that the interaction of the Hat1p/Hat2p sub-complex with ORC occurs preferentially with ORC molecules that are not bound to genomic *ARS *sequences but rather free in the nucleus or perhaps even in the cytoplasm? Indeed, as ORC molecules were found in approximately 10-fold excess over origins [43], one might suggest an overabundance of non-DNA bound ORC in the cell. However, biochemical fractionation experiments in *S. cerevisiae *have clearly demonstrated that ORC, in contrast to the MCM complex, is constitutively chromatin-bound [54]. Therefore, we rather expect that Hat1p/Hat2p-ORC localizes to the nucleus and is probably associated with chromatin (Figure 9).

How could histone H4 acetylation by Hat1p modify ORC and replication origins? Hat1p-mediated acetylation of histone H4 could facilitate the recruitment of additional replication initiation cofactors, such as Cdc45p or the MCM complex, or stimulate the intrinsic ATPase activity of one or more of the ORC subunits, resulting in more extensive remodeling of the chromatin structure at origins. Conversely, ORC-binding could alter the specificity of Hat1p to modify substrates different from free histone H4. Alteration of acetylation specificity by additional acetyltransferase subunits has been shown for the Gcn5p acetyltransferase, which requires additional subunits of the SAGA and Ada2/3 complexes to acetylate nucleosomal substrates [49]. Although we detected no acetylation of nucleosomal substrates by Hat1p/Hat2p-ORC so far (data not shown), Hat1p at origins could acetylate nucleosomes that are transiently unfolded or histones that are exposed in some other way at the onset of DNA replication. Given the numerous reports for non-histone substrates for different acetyltransferases [55], it is also tempting to speculate that Hat1p mediates acetylation of core DNA replication factors.

How can the *orc5-1 hat1 *double mutant phenotypes and the existence of the Hat1p/Hat2p-ORC complex be reconciled with the apparent absence of a direct effect on initiation of DNA replication at the origins that analyzed so far? First, it is formally possible that there are, indeed, real, but subtle defects in the efficiency and timing at chromosomal origins that are difficult to detect by current methodologies. Second, although the Hat1/2p-ORC interaction is clearly separable from the previously documented Hif1p-dependent chromatin assembly complex, it is possible that the *orc5-1 hat1 *phenotype is caused by an indirect problem in chromatin-assembly without affecting replication per se. Such a defect may not necessarily be restricted to origins, and may occur at neighboring sequences. For example, while ORC could recruit Hat1p/Hat2p, the subsequent association with Hif1p could facilitate chromatin assembly at the replication fork. Right now, our data are not sufficient to prove or disprove such a possibility. As a third possibility, it could also be that a constitutive association between Hat1p/Hat2p and ORC occurs only at a subset of origins not examined in this study. This scenario would be reminiscent of the previously reported interaction of ORC with Sum1p, a repressor of mid-sporulation genes. Similar to *hat1Δ *and *hat2Δ*, *sum1Δ *null mutations also reduce the restrictive temperature of *orc-ts *mutations, while having no obvious viability defect on their own [11,56]. Sum1p and ORC also physically interact and, importantly, the chromosomal binding sites of Sum1p show overlap with a subset of replication origins [56,57]. Indeed, Sum1p specifically promoted the activity of the origins to which it was bound. In contrast to Sum1p, however, constitutive binding sites for Hat1p/Hat2p at or near origins would need to be established by more extensive chromatin localization experiments. Nevertheless, the example of the Sum1p-ORC interaction supports the view that different *ARSs *have distinctly different requirements for optimal ORC function, perhaps reflecting different chromatin contexts surrounding these origins. According to this view, Hat1p as a means of modulating local chromatin organization may be just one mechanism among multiple redundant pathways that dictate initiation timing and efficiency at particular origins. Fourth, it is plausible that the Hat1p/Hat2-ORC interaction manifests itself as an alternate function of ORC that is independent from DNA replication. The most obvious candidate for this could be the established transcriptional silencing function of ORC [8,48]. However, the fact that association of Hat1p with ORC persists when the N-terminal silencing domain of Orc1p is deleted (Additional file 5) argues against a connection to the silencing function of ORC. Furthermore, ORC has recently been shown to be involved in the establishment of sister chromatid cohesion in *S. cerevisiae *[10,11]. However, we could not detect defects on sister chromatid cohesion in *hat1Δ *strains (data not shown). While we cannot formally exclude that potential cohesion effects may be very subtle and localized and therefore escape detection, the absence of phenotypes (i.e. plasmid loss, cell-cycle progression) does not support a direct involvement of Hat1p in sister chromatid cohesion either. If constitutive binding sites for Hat1p/Hat2p at origins were identified, a localized role of Hat1p in sister chromatid cohesion may be revealed as well.

The presence of some residual HAT activities associated with ORC in the absence of Hat1p is highly suggestive of potential overlapping functions of HATs (Figure 8). Genetic evidence supports that Hat1p is not the only relevant HAT enzyme with respect to DNA replication. An initial strong candidate for a functionally overlapping *HAT *was Esa1p, which is an essential subunit of the NuA4 complex that specifically acetylates histone H4 and which is responsible for the bulk of histone H4 acetylation in yeast [50,58]. Esa1p has been found to regulate the G1 to S-phase transition [59]. Moreover, a genetic link of NuA4 components with *HAT2 *has been suggested [60]. As metazoan replication origins are less well defined than their yeast counterparts, being more flexible and changing during development, the contribution to epigenetic factors, including histone acetylation, on proper origin activity may be more pronounced in these organisms.

## Conclusion

Physical and genetic interaction data revealed that the yeast B-type histone acetyltransferase Hat1p/Hat2p forms a distinct sub-complex with ORC, providing a potential link between histone acetylation and DNA replication. The evidence presented in this work suggests that this function of Hat1p/Hat2p is distinct from Hif1p-mediated chromatin assembly. As both the B-type histone aceyltransferase and ORC are evolutionary well conserved, we expect that our findings could also be relevant for future investigations in higher eukaryotes.

## Methods

### Yeast strains

Strains used for genetic analysis, TAP-tag, and chromatin immunoprecipitation are listed in Additional file 7. For assays, the haploid strain JRY5128, containing *hat1::HIS3*, was crossed to JRY4250 (*orc5-1*) in order to obtain BSY528 (*hat1::HIS3*) and BSY538 (*orc5-1 hat1::HIS3*). In addition, integrative *hat1Δ::kanMX4 *(BSY539) or *hat2Δ::kanMX4 *(BSY540) knock out alleles were generated by PCR from selected strains in the yeast deletion collection. The *hat1Δ *or *hat2Δ *alleles were also crossed with JRY4125 (*orc2-1*) and JRY6413 (*orc1-161*; obtained from O Aparicio, University of Southern California). TAP- or 13myc-tagged versions of ORC subunits and Hat1p were generated from template plasmids pBS1579 and pFA6-13 myc-kanMX6, respectively. Crosses and transformations were performed according to standard yeast methodologies.

Experiments with histone mutations were performed using strains based on UCC1111 (obtained from M Parthun, Ohio State University). Strains UCC1111 and MPY1 (UCC1111 *hat1Δ::HIS3*) were transformed with a PCR product that contains the *orc5-1 *allele linked to the natMX4 resistance marker to generate AP121 (*orc5-1::natMX4*) and AP123 (*orc5-1::natMX4 hat1Δ::HIS3*). For plasmid shuffling assays, strains AP121 and AP123 were transformed with plasmid containing mutant histone genes (on pMP plasmids). The UCC1111 strains and the pMP plasmids were provided by M Parthun's laboratory [29]. Transformed cells were plated on synthetic complete (SC) -TRP +ADE medium at 23°C to select for the *TRP1 *marked pMP (plasmid), but not for the *ADE2 *marked plasmid. Transformations were performed with pMP3 *HHF2*(WT)-*HHT2*(WT), pMP8 *HHT2*(K9, 14, 18, 23, 27R), pmp48 *HHT2*(K9, 18, 23, 27R), pMP83 *HHT2*(K14, 23R), pMP110 *HHF2*(K5, 12R), and pMP128 *HHF2*(K5, 8, 12, 16R). After 1 week of incubation, transformation plates were scored for white and red colonies. Histone mutants were also introduced in AP182 and AP183 (*hat1Δ::HIS3*) that are *Mat***a **strains derived from UCC1111 by mating-type conversion with HO-endonuclease and with *adh4::URA3::TEL*(*VII-L*) replaced by *adh4::LYS2::TEL*(*VII-L*) using marker swap plasmid M2660 (*ura3::LYS2*).

### Protein purification and Western blots

TAP-tagged proteins were purified as described previously [39]. Purified protein preparations were analyzed both by gel-free tandem mass spectrometry and by gel electrophoresis in conjunction with matrix-assisted laser desorption/ionization-time of flight (MALDI-TOF) mass spectrometry [37]. Raw data and information on the scoring are available from [61].

For co-immunoprecipitation experiments, strains containing myc-tagged Hat1p or Orc5p were used. B Stillman, Cold Spring Harbour Laboratory, NY). Briefly, 50 ml of cells (A_600 _= 1.0) were collected and washed in 1 ml H_2_O. Pellets were resuspended in 200–300 μl modified extraction buffer (50 mM HEPES pH 7.8, 200 mM KCl, 1 mM Na_2_EDTA, 5 mM EGTA-KOH, 10% glycerol, 1 mM DTT) with protease inhibitor cocktail (Roche Diagnostics, Mannheim, Germany) and lysed using a bead beater with 0.4 ml glass beads per sample. Lysates were centrifuged at 14000 rpm for 5 min and the supernatants (whole cell extracts, WCE) were precleared with 20 μl of protein G sepharose beads for 1–2 h. Immunoprecipitation was performed by incubating the precleared extract with 1.5 μl of α-ORC3 antibody (provided by B. Stillman) and 20 μl of protein G beads for 1–3 h. Beads were washed five times with 1 ml extraction buffer and samples were finally resuspended in 10 μl 2× SDS sample buffer before loading on a 10% SDS gel. Western blotting for 13 myc-tagged proteins was performed by using α-myc monoclonal 9E10 (Sigma) as primary antibody (1:5000 dilution) followed by detection with α-mouse IgG horseradish peroxidase (GE Healthcare, Buckinghamshire, UK). Western blot for acetylated H4 lysine 12 was performed using a specific anti-acetyl histone H4 (Lys12) antibody (1:500 dilution; Upstate, Lake Placid, NY, USA), followed by α-rabbit IgG secondary antibody. Hat1p was detected by antibody against Hat1p (yN-20, 1:200 dilution, Santa Cruz CA, USA), followed by α-goat IgG. ORC subunits were probed with by α-ORC3 and α-ORC5 (SB3 and SB5, 1:5000 dilution) and α-ORC2 (SB46; 1:500 dilution; Research Diagnostics, Flanders, NJ, USA), followed by α-mouse IgG horseradish peroxidase.

### Chromatin immunoprecipitation

ChIP assays were performed as described earlier [22] Strains were incubated in YPD media until they reached early log phase (A_600 _of approximately 0.5). Cells were arrested in 5 μg/ml α-factor for 2.5 h at 30°C or 36°C (*orc2-1 *and *cdc7-1 *inactivation), and were released into fresh medium at 16°C/23°C or 36°C Samples were taken for analysis at indicated time points. Chromatin was sheared by sonication to the average fragment size of 0.2–1 kB (as determined by agarose gel electrophoresis). For anti-TAP IPs, 20 μl of IgG sepharose beads were incubated with 2–3 μg of total protein in an immunoprecipitation volume of 400 μl for 2 h at 4°C. After extensive washing, DNA was eluted from beads, de-crosslinked and purified using QIAGEN PCR purification kit.

PCR reactions were carried out in a final volume of 12 μl. A total of 1–4% of immunoprecipitated material and 0.01–0.04% of input material were added to each tube followed by 1.2 μl of each primer (10 μM stock), 1.2 μl of 2.5 mM dNTP mix, 1.2 μl of 10× PCR buffer, 0.06 μl [^32^P]dATP (10 mCi/ml) and 0.25 μl of Expand (Roche). PCR parameters were: 94°C, 2 min (94°C, 30 s; 55°C, 30 s; 72°C, 1 min) × 27 72°C, 4 min. PCR products were resolved in 6% polyacrylamide/1× TBE gels. After the gels were dried, a PhosphorImager (Storm; Molecular Dynamics, Sunnyvale, CA) was used for scanning and quantification. Alternatively, PCR was performed without radioactive label and PCR products were separated on 1.5% agarose gels and stained in ethidium bromide. For quantification, signals from the immunoprecipitates were divided by the corresponding input signals, correcting for the dilution factor. For primers for chromatin immunoprecipitations see Additional file 8.

### HAT activity assay

HAT assays were essentially performed as described before [62]. TAP-purified proteins were concentrated approximately 10-fold using Vivaspin 5000 columns (Sartorius AG, Goettingen, Germany,). Reactions were performed using 1 μl concentrated enzyme, 5 μg purified chicken core histones, 0.1 μl ^14^C-acetyl-coenzyme A (AcCoA) (50 mCi/mmol) in 15 μl 1 × buffer (50 mM sodium phosphate pH 7.4, 15 mM 2-mercaptoethanol, 10% glycerol) for 15 min at 37°C. Histones were separated on 15% SDS-PAGE and visualized by Coomassie staining. Gels were exposed, scanned using a PhosphorImager and quantified using the IQMac v. 1.2 software (Molecular Dynamics, Sunnyvale, CA). The amount of ^14^C incorporation was determined according to a separate standard of ^14^C-histones. Preparation of chicken core histones and nucleosomes was performed as described previously [63].

### Cell-cycle analysis and viability assays

For cell-cycle analysis under semi-permissive conditions, exponentially growing cells were synchronized in 5 μg/ml α-factor at A_600 _= 0.2 for 2–2.5 h at 31°C for *orc5-1 *and at 26°C for *orc2-1*. For cell-cycle analysis under restrictive conditions, exponentially growing cells were synchronized in 5 μg/ml α-factor at A_600 _= 0.2 for 2 or 2.5 h at 23°C and then shifted to 36°C with an additional 5 μg/ml α-factor. Release was then performed in fresh YPD medium. Fluorescence activated cell sorting (FACS) analysis of synchronized cultures was performed by SYTOX staining (SYBr; Molecular Probes, Eugene, OR). Cells (1 ml) were harvested at indicated timepoints and fixed in 70% EtOH. The fixed cells were washed in 1 × PBS and sonicated for 10 s. Cells were then incubated overnight in 1 × PBS containing 0.25 mg/ml RNaseA at 37°C. After RNase, cells were incubated for 1 h at 37°C in 0.2 ml of 5 mg/ml Pepsin solution and then stored in 1 × PBS. Diluted cells were stained in 1 ml 1 × PBS containing 1 × SYTOX for 1 h, and DNA content was determined with a Becton Dickinson FACScan) or a FACScalibur flow cytometer (BD Biosciences, Mountain View, CA). A total of 10000 cells were counted per sample. Alternatively, staining was also performed in 16 μg/ml propidium iodide.

For cell-cycle dependent loss of viability assays, cells were either arrested in G1-phase with 5 μg/ml α-factor or in S-phase with 200 mM hydroxyurea at 23°C for 2.5 h and then shifted for 3 h at 36°C. When cells were kept in G1, an additional 5 μg/ml α-factor was added. Dilutions of 1/1000 were plated on YPD plates and the fraction of viable microcolonies from total plated cells was analyzed after 1 or 2 days at 23°C.

### Plasmid loss and analysis of replicative intermediates

Calculation of plasmid loss rate has been described [9]. Plasmid loss rates were measured in *orc5-1 hat1*, *orc5-1, hat1*, and wild-type control strain that contain plasmids pDK243, pDK368-1, pDK368-2, and pDK368-7. pDK243 contains one *ARS1 *sequence. pDK368-1, pDK368-2, and pDK368-7 contain additionally 1, 2 and 7 *ARSH4 *sequences. Dilutions were taken from cultures after 0 or approximately 10 generations in nonselective growth conditions and plated on SC medium. Grown colonies were replica-plated on SC -Leu medium and the plasmid loss rate was calculated from the fraction of cells that retained the *LEU2 *marked plasmids. For determination of loss rates of *URA3 *containing plasmids YCp120 and pRS316, the replica plating was performed on -URA medium.

For analysis of replicative intermediates, cells were synchronized at A_600 _= 0.3–0.4 in 3 μg/ml or 5 μg/ml α-factor for 2 h and released into fresh medium at 30°C. Samples (250 ml) were taken at 15 and 30 min after release from α-factor arrest and treated with 2.5 ml NaN_3_. DNA extraction by CTAB lysis and two-dimensional gel electrophoresis were performed as described previously [46]. DNA was digested with *Nco*I into a 5.1 kB fragment for *ARS305 *and a 4.4 kB fragment for *ARS1 *detection. The signals from the Southern blots were scanned by PhosphorImager and calculated by the IQMac v1.2 program.

## Abbreviations

AcCoA, Acetyl-Coenzyme A; *ARS*, Autonomous Replicating Sequence; CBP, Calmodulin Binding Protein; CTAB, Cetyltrimethylammonium Bromide; FACS, Fluorescence Activated Cell Sorting; HAT, Histone Acetyltransferase; MALDI-TOF, Matrix-assisted Laser Desorption/Ionization – Time of Flight; LC-MS, Liquid Chromatography – Mass Spectrometry; MCM, Minichromosome Maintenance; ORC, Origin Recognition Complex; PBS, Phosphate Buffered Saline; pre-RC, pre-Replicative Complex; SC, Synthetic Complete; SGA, Synthetic Genetic Array; TAP, Tandem Affinity Purification; YPD, Yeast Peptone Dextrose.

## Competing interests

The author(s) declares that there are no competing interests.

## Authors' contributions

BS, OP and XG carried out all experimental procedures. XG, NK, and JG were responsible for the TAP protein purifications. PL supported and provided materials for the histone acetylation assays. JR supported the genetic analysis and the execution of the study. BS and AE designed the experiments and wrote the manuscript. All authors approved the final manuscript.

## Supplementary Material

Additional file 1 **Histone acetylation by Hat1p sub-complexes and Hat1p-ORC interaction **(A) Silver stain gel of additional TAP purifications that were used for the Western blot in Figure 1D (left panel). Strains are *HAT1-TAP *(BSY679), *HAT1-TAP hat2Δ *(BSY682), and *HAT1-TAP hif1Δ *(BSY720). (B) Quantification of *in vitro *histone acetyltransferase activities of Hat1p complexes in Figure 1E. Decays per min are shown on the Y-axis. (C) Immunoprecipitation using the a-ORC3 antibody was performed as described in Figure 2 except that the samples were split in two and half was treated with benzonase. After immunoprecipitation, protein G beads were treated with 90 units of benzonase (Novagen) for 30 min on ice in a volume of 120 ml buffer (20 mM Tris-HCl pH 7.5, 2 mM MgCl_2_, 20 mM KCl, 10% glycerol). Protein G beads were washed two times before and after digestion with benzonase buffer. Immunoprecipitated Orc5–13 myc, Hat1–13 myc (α-myc primary antibody), and Orc3 (α-ORC3 antibody) is shown. Click here for file

Additional file 2 **Combination of *hat1Δ *with *mcm2-1 *and *cdc7-1***. (A) Plating assay of *mcm2-1 hat1Δ *(BSY032, BSY033) and control strains at indicated temperatures. Dilutions were 1:10, starting from late log-phase cultures in YPD. (B) Combination of *hat1Δ *with the *cdc7-1 *(JRY4553) allele. The *cdc7-1 hat1Δ *mutant (BSY619) and control strains were grown at semipermissive temperature (26°C) for 3 days. For strains, see Additional file 7. Click here for file

Additional file 3 **The effect of *hat1Δ *and *hat2Δ *on cell-cycle progression**. (A) Cell-cycle progression of *orc5-1 hat11Δ *(BSY617) and control strains (BSY616, JRY2334) at semi-permissive temperature (31°C). Cells were arrested in G1 and then released for the timepoints indicated (0, 20, 30, 40, 60, 90, 120, 180, and 300 min). Peak positions of G1 and G2 cells are indicated. (B) FACS analysis in *hat2Δ *(BSY550) and wild-type (BSY551) cells. Cells were arrested in G1 and release was performed at 30°C for 0, 10, 20, 25, 30, 40, 50, 60, 70, and 80 min. (C) Cell-cycle progression of *orc5-1 hat1Δ *is affected by the DNA damage checkpoint. Cells were arrested in G1 and released at 31°C for 0, 10, 20, 30, 40, 50, 60, 90, 120, and 180 min. Strains were *orc5-1 *(BSY614), *orc5-1 hat1Δ *(BSY615), *orc5-1 rad9 rad24 *(BSY613), and *orc5-1 hat1Δ rad9 rad24 *(BSY612). See Additional file 7 for detailed description of strains. Click here for file

Additional file 4 **Structural analysis of replicative intermediates by two-dimensional gel electrophoresis**. Cultures (*orc5-1 hat1*, *orc5-1*, wild-type) were released from G1 arrest for 15 and 30 min (30°). Strains were JRY2334 (*W303 *wild-type control), BSY535 (*orc5-1*), and BSY538 (*orc5-1 hat1*). The same blot was first hybridized with an *ARS305 *specific probe (A), then stripped and rehybridized with an *ARS1 *specific probe (B). Note that *ARS305 *seems to be more resistant to the effect of the *orc5-1 *mutation than *ARS1*. Click here for file

Additional file 5 **The Hat1p/Hat2p-ORC interaction is not dependent on the N-terminal silencing domain of Orc1p**. (A) Immunoprecipitations with α-Orc3 antibody from extracts of Hat1–13myc-tagged *ORC1 *(BSY679), N-terminal deletion of *ORC1 *(*ORC1Δ1–235*, BSY702), N-terminal fusion of *SIR3 *with *ORC *(*SIR3N*/*ORC1*, BSY703), and untagged wild-type strains are shown along with whole cell extracts. Immunoprecipiations were performed either with αORC3 or αGFP control antibody. (B) Mating test of Mata wild-type *ORC1*, *ORC1Δ1–235*, and *SIR3N*/*ORC1 *strains, containing *HIS3::HMRα*(-*rap1*). Click here for file

Additional file 6 **Growth test of strains containing nonacetylatable N-terminal mutations**. (A) Comparison of UCC1111 and AP121 (*orc5-1*) containing wild-type histone H3 and H4 (pmp3), K9, 14, 18, 23, 27R substituted histone H3 (pmp8), and K5, 12R substituted histone H4 (pmp110). Plate was incubated at 30°C for 3 days. (B) Comparison of AP121 (*orc5-1*) and AP123 (*orc5-1 hat1Δ*) containing wild-type histone H3 and H4 (pmp3), K5, 12R substituted histone H4 (pmp110), K5, 8, 12, 16R substituted histone H4 (pmp128), and K14, 23R substituted histone H3 (pmp83). **(C) **Growth test for strains using in plasmid loss assays. Strains AP182 and AP183 containing wild-type histone H3 (pmp3), K9, 14, 18, 23, 27R substituted histone H3 (pmp8), and K9, 14R substituted histone H3 (pmp83) at 30°C for 3 days. Click here for file

Additional file 7 Strains used for genetic analysis, TAP-tag, and chromatin immunoprecipitation Click here for file

Additional file 8 Primers used for chromatin immunoprecipitation Click here for file
