# Supplementary figures and images for: Association with the origin recognition complex suggests a novel role for histone acetyltransferase Hat1p/Hat2p
Source: BMC Biol. 2007 Sep 19;5:38. doi: 10.1186/1741-7007-5-38 (PMC2140264; doi:10.1186/1741-7007-5-38)

**A**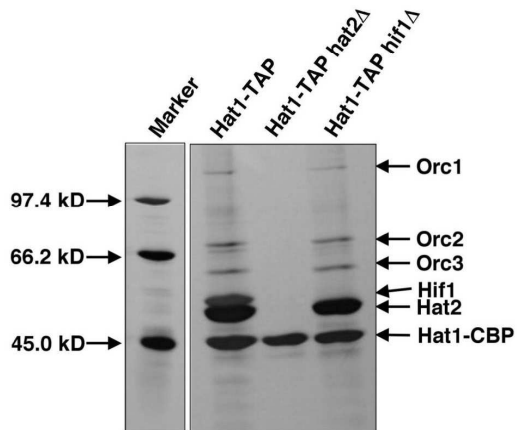**B**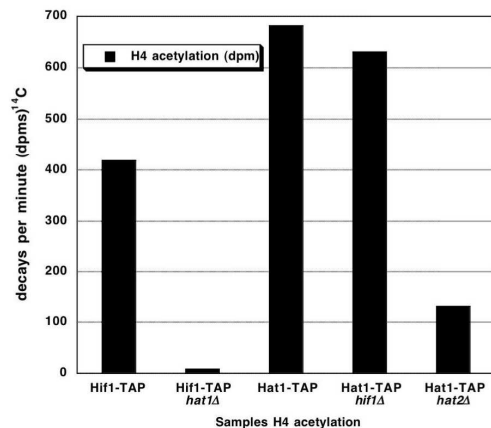**C**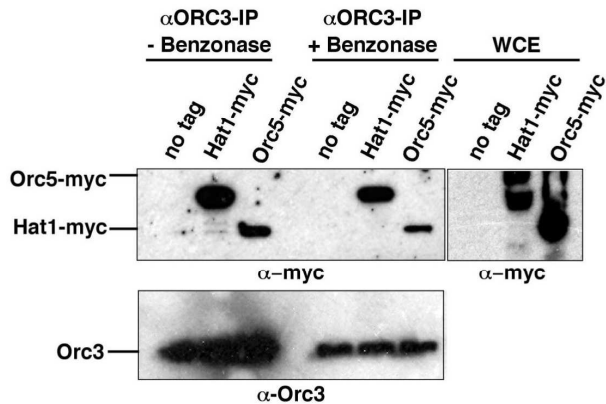

Supplement: Additional file 1 — Histone acetylation by Hat1p sub-complexes and Hat1p-ORC interaction (A) Silver stain gel of additional TAP purifications that were used for the Western blot in Figure 1D (left panel). Strains are HAT1-TAP (BSY679), HAT1-TAP hat2Δ (BSY682), and HAT1-TAP hif1Δ (BSY720). (B) Quantification of in vitro histone acetyltransferase activities of Hat1p complexes in Figure 1E. Decays per min are shown on the Y-axis. (C) Immunoprecipitation using the a-ORC3 antibody was performed as described in Figure 2 except that the samples were split in two and half was treated with benzonase. After immunoprecipitation, protein G beads were treated with 90 units of benzonase (Novagen) for 30 min on ice in a volume of 120 ml buffer (20 mM Tris-HCl pH 7.5, 2 mM MgCl2, 20 mM KCl, 10% glycerol). Protein G beads were washed two times before and after digestion with benzonase buffer. Immunoprecipitated Orc5–13 myc, Hat1–13 myc (α-myc primary antibody), and Orc3 (α-ORC3 antibody) is shown. [file 1741-7007-5-38-S1.pdf]

**A**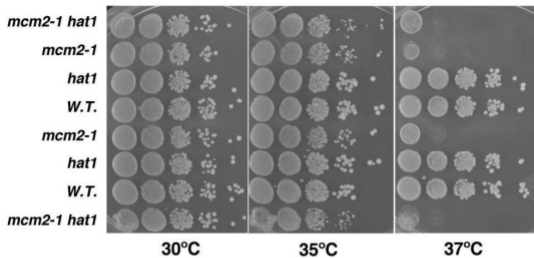**B**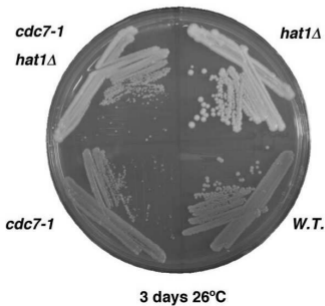

Supplement: Additional file 2 — Combination of hat1Δ with mcm2-1 and cdc7-1. (A) Plating assay of mcm2-1 hat1Δ (BSY032, BSY033) and control strains at indicated temperatures. Dilutions were 1:10, starting from late log-phase cultures in YPD. (B) Combination of hat1Δ with the cdc7-1 (JRY4553) allele. The cdc7-1 hat1Δ mutant (BSY619) and control strains were grown at semipermissive temperature (26°C) for 3 days. For strains, see Additional file 7. [file 1741-7007-5-38-S2.pdf]

**A**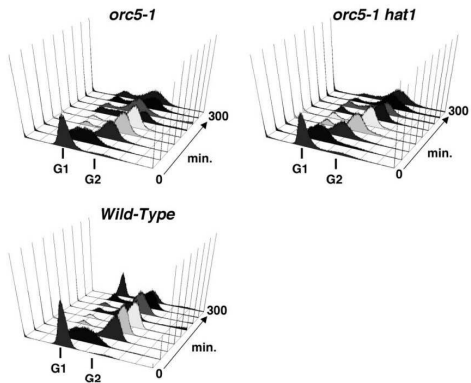**B**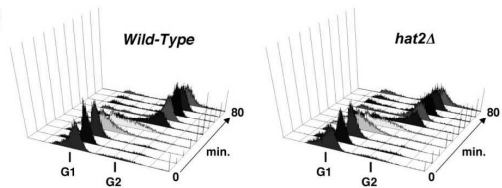**C**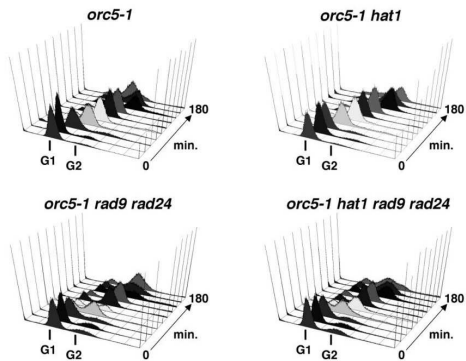

Supplement: Additional file 3 — The effect of hat1Δ and hat2Δ on cell-cycle progression. (A) Cell-cycle progression of orc5-1 hat11Δ (BSY617) and control strains (BSY616, JRY2334) at semi-permissive temperature (31°C). Cells were arrested in G1 and then released for the timepoints indicated (0, 20, 30, 40, 60, 90, 120, 180, and 300 min). Peak positions of G1 and G2 cells are indicated. (B) FACS analysis in hat2Δ (BSY550) and wild-type (BSY551) cells. Cells were arrested in G1 and release was performed at 30°C for 0, 10, 20, 25, 30, 40, 50, 60, 70, and 80 min. (C) Cell-cycle progression of orc5-1 hat1Δ is affected by the DNA damage checkpoint. Cells were arrested in G1 and released at 31°C for 0, 10, 20, 30, 40, 50, 60, 90, 120, and 180 min. Strains were orc5-1 (BSY614), orc5-1 hat1Δ (BSY615), orc5-1 rad9 rad24 (BSY613), and orc5-1 hat1Δ rad9 rad24 (BSY612). See Additional file 7 for detailed description of strains. [file 1741-7007-5-38-S3.pdf]

**A**

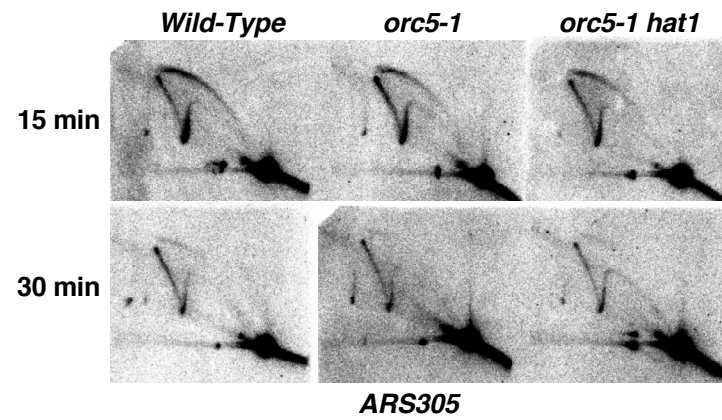

**B**

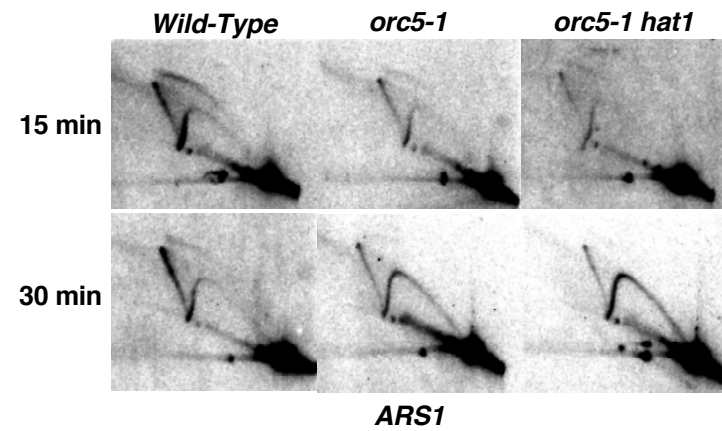

**Additional figure 4.**

Supplement: Additional file 4 — Structural analysis of replicative intermediates by two-dimensional gel electrophoresis. Cultures (orc5-1 hat1, orc5-1, wild-type) were released from G1 arrest for 15 and 30 min (30°). Strains were JRY2334 (W303 wild-type control), BSY535 (orc5-1), and BSY538 (orc5-1 hat1). The same blot was first hybridized with an ARS305 specific probe (A), then stripped and rehybridized with an ARS1 specific probe (B). Note that ARS305 seems to be more resistant to the effect of the orc5-1 mutation than ARS1. [file 1741-7007-5-38-S4.pdf]

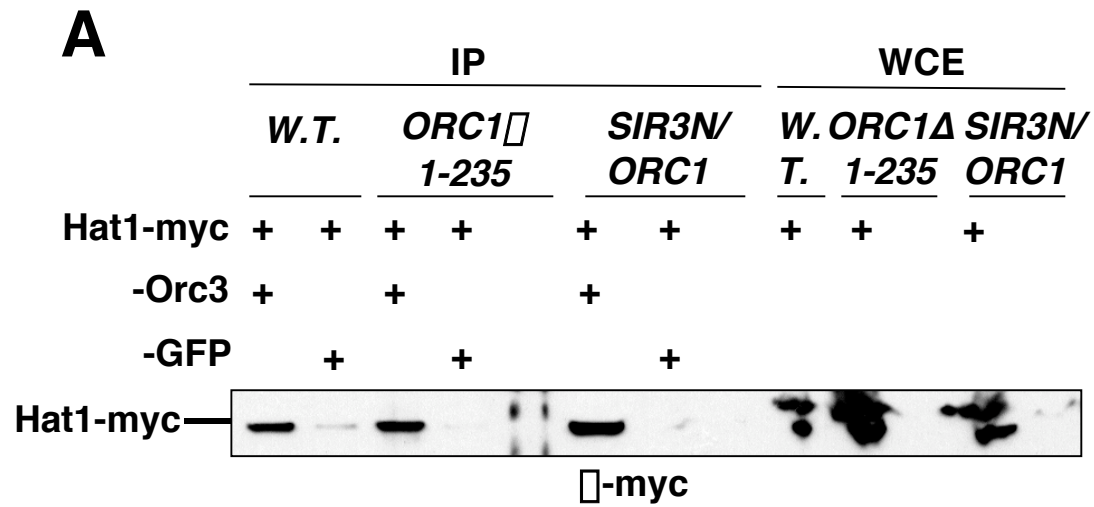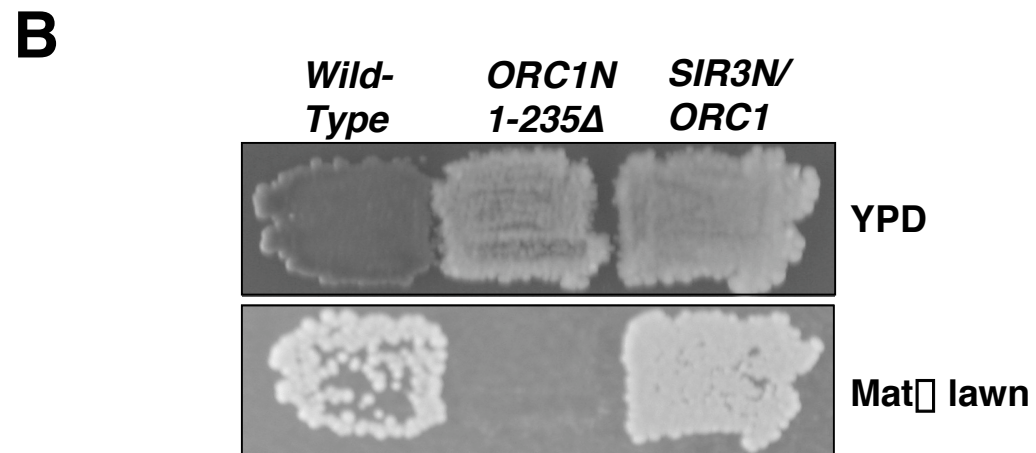

Additional figure 5.

Supplement: Additional file 5 — The Hat1p/Hat2p-ORC interaction is not dependent on the N-terminal silencing domain of Orc1p. (A) Immunoprecipitations with α-Orc3 antibody from extracts of Hat1–13myc-tagged ORC1 (BSY679), N-terminal deletion of ORC1 (ORC1Δ1–235, BSY702), N-terminal fusion of SIR3 with ORC (SIR3N/ORC1, BSY703), and untagged wild-type strains are shown along with whole cell extracts. Immunoprecipiations were performed either with αORC3 or αGFP control antibody. (B) Mating test of Mata wild-type ORC1, ORC1Δ1–235, and SIR3N/ORC1 strains, containing HIS3::HMRα(-rap1). [file 1741-7007-5-38-S5.pdf]

**A**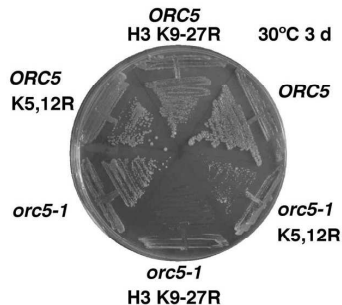**B**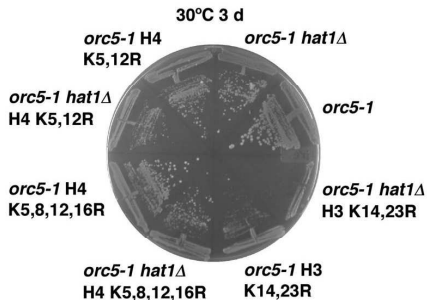**C**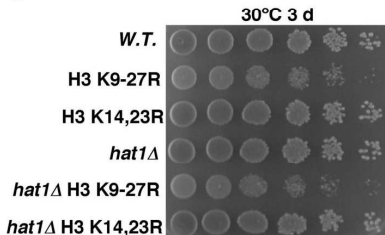

Supplement: Additional file 6 — Growth test of strains containing nonacetylatable N-terminal mutations. (A) Comparison of UCC1111 and AP121 (orc5-1) containing wild-type histone H3 and H4 (pmp3), K9, 14, 18, 23, 27R substituted histone H3 (pmp8), and K5, 12R substituted histone H4 (pmp110). Plate was incubated at 30°C for 3 days. (B) Comparison of AP121 (orc5-1) and AP123 (orc5-1 hat1Δ) containing wild-type histone H3 and H4 (pmp3), K5, 12R substituted histone H4 (pmp110), K5, 8, 12, 16R substituted histone H4 (pmp128), and K14, 23R substituted histone H3 (pmp83). (C) Growth test for strains using in plasmid loss assays. Strains AP182 and AP183 containing wild-type histone H3 (pmp3), K9, 14, 18, 23, 27R substituted histone H3 (pmp8), and K9, 14R substituted histone H3 (pmp83) at 30°C for 3 days. [file 1741-7007-5-38-S6.pdf]
