# Supplementary material for: Association with the origin recognition complex suggests a novel role for histone acetyltransferase Hat1p/Hat2p
Source: BMC Biol. 2007 Sep 19;5:38. doi: 10.1186/1741-7007-5-38 (PMC2140264; doi:10.1186/1741-7007-5-38)
Supplement: Additional file 7 — Strains used for genetic analysis, TAP-tag, and chromatin immunoprecipitation [file 1741-7007-5-38-S7.pdf]

Additional table 1. Yeast strains used in this study.

Strain	Genotype	Source[a]
JRY2334	W303-1a *Mat***a** *ade2-1 leu2-112 his3-11,15 trp1-1 ura3-1*	R. Rothstein
JRY4125	*Matα ade2-1 leu2-112 his3-11,15 trp1-1 ura3-1 orc2-1*	
JRY4250	*Matα ade2-1 leu2-3,112 his3-11,15 trp1-1 ura3-1 orc5-1*	
JRY4553	*Mat***a** *lys2Δ leu2-112 his3-11,15 trp1-1 ura3-1 cdc7-1*	
JRY5128	*Matα lys2Δ leu2-112 his3-11,15 trp1-1 ura3-1 hat1::HIS3 hmrΔ::URA3*	
JRY6413	*Mat***a** *bar1::hisG lys2::hisG his3-11,15 leu2-3,112 trp1-1 ura3-1 orc1::hisG leu2::orc1-161-LEU2*	S. Bell
PGY974	*Matα ade2-1 leu2-112 his3-11,15 trp1-1 ura3-1 mcm2-1*	P. Garber
UCC1111	*Matα ade2::hisG his3Δ200 leu2Δ0 lys2Δ0 met15Δ0 trp1Δ63 ura3Δ0 adh4:::URA3-TEL(VII-L) hhf1-hht1::LEU2 hhf2-hht2::MET15 pRS412 (ADE2 CEN ARS – HHF2(WT)-HHT2(WT))*	M. Parthun
MPY1	UCC1111 *hat1::HIS3*	M. Parthun
AP121	UCC1111 *orc5-1::natMX4*	
AP123	UCC1111 *orc5-1::natMX4 hat1::HIS3*	
AP182	UCC1111 *Mat***a** *adh4::LYS2-TEL(VII-L)*	

AP183	UCC1111 *Mat***a** *adh4::LYS2-TEL(VII-L) hat1::HIS3*	
YSB0133	*Mat***a** *ade2-1 leu2-112 his3-11,15 trp1-1 ura3-1 orc1::TRP1 LEU2::ORC1 (Δ1-235) HIS3::HMRα(-RAP1)*	S. Bell
YSB0135	*Mat***a** *ade2-1 leu2-112 his3-11,15 trp1-1 ura3-1 orc1::TRP1 LEU2::ORC1 (Δ1-235)/SIR3 HIS3::HMRα(-RAP1)*	S. Bell
BSY029	UCC1111 *pMP3 [pRS314 TRP1 ARS – HHF2 (WT)-HHT2(WT)]*	
BSY030	UCC1111 *pMP8 [pRS314 TRP1 ARS – HHF2 (WT)-HHT2(K9, 14, 18, 23, 27R*)]	
BSY031	UCC1111 *pMP110 [pRS314 TRP1 ARS – HHF2 (K5, 12R)-HHT2(WT)]*	
BSY032	*Matα ade2-1 leu2-112 his3-11,15 trp1-1 ura3-1 mcm2-1 hat1::HIS3*	
BSY033	*Mat***a** *ade2-1 leu2-112 his3-11,15 trp1-1 ura3-1 mcm2-1 hat1::HIS3*	
BSY044	BSY029 *orc5-1::natMX4*	
BSY045	BSY030 *orc5-1::natMX4*	
BSY070	BSY031 *orc5-1::natMX4*	
BSY528	*Mat***a** *ade2-1 leu2-112 his3-11,15 trp1-1 ura3-1 hat1::HIS3*	
BSY535	*Mat***a** *ade2-1 leu2-112 his3-11,15 trp1-1 ura3-1 orc5-1*	

BSY538	*Mat***a** *ade2-1 leu2-112 his3-11,15 trp1-1 ura3-1 orc5-1 hat1::HIS3*
BSY539	*Mat***a** *ade2-1 leu2-112 his3-11,15 trp1-1 ura3-1 hat1Δ::kanMX4*
BSY540	*Mat***a** *ade2-1 leu2-112 his3-11,15 trp1-1 ura3-1 hat2Δ::kanMX4*
BSY550	*Mat***a** *ade2-1 leu2-112 his3-11,15 trp1-1 ura3-1 hat2Δ::kanMX4 bar1::HIS3*
BSY551	*Mat***a** *ade2-1 leu2-112 his3-11,15 trp1-1 ura3-1 bar1::HIS3*
BSY568	*Mat***a** *ade2-1 leu2-112 his3-11,15 trp1-1 ura3-1 orc2-1*
BSY569	*Mat***a** *ade2-1 leu2-112 his3-11,15 trp1-1 ura3-1 orc2-1 hat1Δ::kanMX4*
BSY572	*Mat***a** *ade2-1 leu2-112 his3-11,15 trp1-1 ura3-1 orc2-1 hat2Δ::kanMX4*
BSY589	*Mat***a** *ade2-1 lys2::hisG his3-11,15 leu2-3,112 trp1-1 ura3-1 orc1::hisG leu2::orc1-161-LEU2*
BSY590	*Matα ade2-1 lys2::hisG his3-11,15 leu2-3,112 trp1-1 ura3-1 orc1::hisG leu2::orc1-161-LEU2*
BSY591	*Mat***a** *ade2-1 his3-11,15 leu2-3,112 trp1-1 ura3-1 orc1::hisG leu2::orc1-161-LEU2*
BSY594	*Mat***a** *ade2-1 LYS2 his3-11,15 leu2-3,112 trp1-1 ura3-1 hif1Δ::kanMX4*
BSY595	*Matα ade2-1 LYS2 his3-11,15 leu2-3,112 trp1-1 ura3-1 orc5-1 hif1Δ::kanMX4*
BSY602	*Mat***a** *ade2-1 LYS2 his3-11,15 leu2-3,112 trp1-1 ura3-1 orc5-1 hat2Δ::kanMX4*
BSY603	*Matα ade2-1 lys2Δ his3-11,15 leu2-3,112 trp1-1 ura3-1 orc5-1 hat2Δ::kanMX4 hat1::HIS3*

BSY604	*Mat***a** *ade2-1 his3-11,15 leu2-3,112 trp1-1 ura3-1 orc5-1 hat2Δ::kanMX4 hat1::HIS3*
BSY612	*Mat***a** *ade2-1 his3-11,15 leu2-3,112 trp1-1 ura3-1 orc5-1 hat1Δ::kanMX4 rad9::HIS3 rad24::TRP1*
BSY613	*Mat***a** *ade2-1 his3-11,15 leu2-3,112 trp1-1 ura3-1 orc5-1 rad9::HIS3 rad24::TRP1*
BSY614	*Mat***a** *ade2-1 his3-11,15 leu2-3,112 trp1-1 ura3-1 orc5-1*
BSY615	*Mat***a** *ade2-1 his3-11,15 leu2-3,112 trp1-1 ura3-1 orc5-1 hat1::kanMX4*
BSY616	*Mat***a** *ade2-1 his3-11,15 leu2-3,112 trp1-1 ura3-1 orc5-1*
BSY617	*Mat***a** *ade2-1 his3-11,15 leu2-3,112 trp1-1 ura3-1 orc5-1 hat1Δ::kanMX4*
BSY619	*Mat***a** *lys2Δ leu2-112 his3-11,15 trp1-1 ura3-1 cdc7-1 hat1Δ::kanMX4*
BSY629	*Mat***a** *his3Δ1 leu2Δ0 met15Δ0 ura3Δ0 ORC1-TAP::TRP1*
BSY630	*Mat***a** *his3Δ1 leu2Δ0 met15Δ0 ura3Δ0 ORC2-TAP::TRP1*
BSY631	*Mat***a** *his3Δ1 leu2Δ0 met15Δ0 ura3Δ0 ORC4-TAP::TRP1*
BSY632	*Mat***a** *his3Δ1 leu2Δ0 met15Δ0 ura3Δ0 ORC5-TAP::TRP1*
BSY633	*Mat***a** *his3Δ1 leu2Δ0 met15Δ0 ura3Δ0 ORC6-TAP::TRP1*
BSY675	*Mat***a** *his3Δ1 leu2Δ0 met15Δ0 ura3Δ0 HIF1-TAP::TRP1*
BSY676	*Mat***a** *ade2-1 LYS2 his3-11,15 leu2-3,112 trp1-1 ura3-1 HAT1-13myc::kanMX6*

BSY677	*Mat***a** *ade2-1 LYS2 his3-11,15 leu2-3,112 trp1-1 ura3-1 ORC5-13myc::kanMX6*
BSY679	*Mat***a** *ade2-1 LYS2 his3-11,15 leu2-3,112 trp1-1 ura3-1 HAT1-TAP::TRP1*
BSY680	*Mat***a** *ade2-1 LYS2 his3-11,15 leu2-3,112 trp1-1 ura3-1 CDC45-TAP::TRP1*
BSY681	*Mat***a** *ade2-1 LYS2 his3-11,15 leu2-3,112 trp1-1 ura3-1 HIF1-TAP::TRP1 hat1Δ::kanMX4*
BSY682	*Mat***a** *ade2-1 LYS2 his3-11,15 leu2-3,112 trp1-1 ura3-1 HAT1-TAP::TRP1 hat2Δ::kanMX4*
BSY691	*Mat***a** *ade2-1 LYS2 his3-11,15 leu2-3,112 trp1-1 ura3-1 HAT2-13myc::kanMX6*
BSY692	*Mat***a** *ade2-1 LYS2 his3-11,15 leu2-3,112 trp1-1 ura3-1 HIF1-13myc::kanMX6*
BSY699	*Mat***a** *ade2-1 LYS2 his3-11,15 leu2-3,112 trp1-1 ura3-1 orc2-1 HAT1-TAP::TRP1*
BSY700	*Mat***a** *ade2-1 leu2-112 his3-11,15 trp1-1 ura3-1 ORC5-TAP::TRP1*
BSY701	*Mat***a** *ade2-1 leu2-112 his3-11,15 trp1-1 ura3-1 ORC5-TAP::TRP1 hat1Δ::natMX4*
BSY702	YSB0133 *HAT1-13myc::kanMX6*
BSY703	YSB0135 *HAT1-13myc::kanMX6*
BSY720	*Mat***a** *ade2-1 leu2-112 his3-11,15 trp1-1 ura3-1 1 HAT1-TAP::TRP1 hif1Δ::natMX4*
BSY734	*Mat***a** *ade2-1 leu2-112 his3-11,15 trp1-1 ura3-1 1 HAT1-TAP::TRP1 cdc7-1*

[a]Strains that were not constructed in our laboratories are indicated.
