# Supplementary material for: Association with the origin recognition complex suggests a novel role for histone acetyltransferase Hat1p/Hat2p
Source: BMC Biol. 2007 Sep 19;5:38. doi: 10.1186/1741-7007-5-38 (PMC2140264; doi:10.1186/1741-7007-5-38)
Supplement: Additional file 8 — Primers used for chromatin immunoprecipitation [file 1741-7007-5-38-S8.doc]

**Additional table 2.** Primers for chromatin immunoprecipitation.

| Primer | Sequence |
| --- | --- |
| ARS1-F | GGTGGACTGACGCCAGAAAATG |
| ARS1-R | GCGGTGAAATGGTAAAAGTCAAC |
| R11-F | CACCGATACGTACTTAAACTCT |
| R11-R | GAGAAAGCTTAGTCCATTCGGC |
| ARS305-F | GATTGAGGCCACAGCAAGACCGG |
| ARS305-R | CTCCGTTTTTAGCCCCCGTGTAAG |
| ARS305+8KB-F | GGTGGTGGAGAAGCGGTTCAAAG |
| ARS305+8KB-R | CCGCTCGTACCCGCTCCTGA |
| ARS1412-F | GTCACTGACCGCGGCTAAAAG |
| ARS1412-R | GCAAGCTCACTCTCTTTGTTC |
